# Parvalbumin- and vasoactive intestinal polypeptide-expressing neocortical interneurons impose differential inhibition on Martinotti cells

**DOI:** 10.1038/ncomms13664

**Published:** 2016-11-29

**Authors:** F. Walker, M. Möck, M. Feyerabend, J. Guy, R. J. Wagener, D. Schubert, J. F. Staiger, M. Witte

**Affiliations:** 1 University Medical Center Göttingen, Institute for Neuroanatomy, Göttingen 37075, Germany; 2 Radboud University Medical Centre Nijmegen, Department of Cognitive Neuroscience, Donders Institute for Brain, Cognition and Behaviour, 6525 EN Nijmegen, Netherlands

## Abstract

Disinhibition of cortical excitatory cell gate information flow through and between cortical columns. The major contribution of Martinotti cells (MC) is providing dendritic inhibition to excitatory neurons and therefore they are a main component of disinhibitory connections. Here we show by means of optogenetics that MC in layers II/III of the mouse primary somatosensory cortex are inhibited by both parvalbumin (PV)- and vasoactive intestinal polypeptide (VIP)-expressing cells. Paired recordings revealed stronger synaptic input onto MC from PV cells than from VIP cells. Moreover, PV cell input showed frequency-independent depression, whereas VIP cell input facilitated at high frequencies. These differences in the properties of the two unitary connections enable disinhibition with distinct temporal features.

## Abstract (web summary)

Martinotti cells disinhibit excitatory cells in the brain cortex and play an important role in information flow. Here the authors study the role of parvalbumin and vasoactive intestinal polypeptide interneurons on the inhibition of Martinotti cells in the mouse somatosensory cortex.

## Graphical abstract

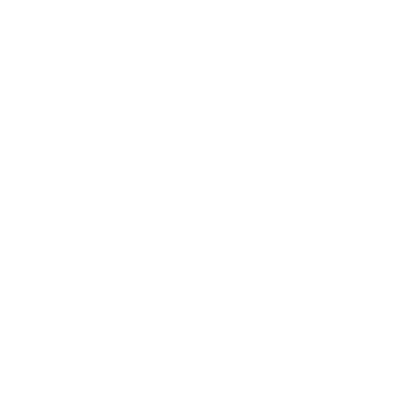

Cortical inhibitory interneurons (IN) are grouped into three major subpopulations defined by the expression of molecular markers, namely parvalbumin (PV), somatostatin (SST) and the ionotropic serotonin receptor (5HT_3a_R)^1^,^2^. The main, but not exclusive, cell types within these subpopulations, largely defined by morphological features, are: PV-expressing basket cells, SST-expressing Martinotti cells (MC) and cells co-expressing 5HT_3a_R and vasoactive intestinal polypeptide (VIP)^1^,^3^,^4^. The functions of these IN are manifold. In general, they keep excitation in check, perform gain modulation and induce synchronization and oscillations, whereas more specifically they open or close temporal or spatial windows for input control or output generation^5^. Their functional impact is not restricted to their interaction with excitatory neurons, but direct interactions between IN seem to be essential for sensory information processing as well^6^,^7^,^8^,^9^.

In the rodent primary somatosensory (barrel) cortex (S1), GABAergic MC in layer (L) II/III^10^ mediate disynaptic inhibition between neighbouring pyramidal cells (PCs) and thereby have a major impact on the flow of information within and across cortical columns^2^,^11^,^12^,^13^. MC in turn are inhibited by other IN, leading to disinhibition of PCs^6^,^7^,^8^,^14^. Several recent *in vivo* studies have shown that such disinhibition via MC (or other types of IN) contributes to sensory-guided behaviour and learning^7^,^14^,^15^. Morphological and physiological differences of presynaptic IN subtypes^16^,^17^ may result in distinct versions of disinhibition in the spatial and/or temporal domain. If so, the regulation of cortical processing by disinhibition might be much more flexible than simply being an on/off switch. Therefore, we aimed to define the molecular identity of IN targeting LII/LIII MC by optogenetics, localize these cells by using glutamate uncaging and study the unitary properties and short-term plasticity of their synaptic transmission by paired recordings. We found that MC in mouse primary somatosensory cortex commonly receive inhibitory inputs from local PV- and VIP-expressing interneurons. Further, these inputs differ substantially in unitary properties and short-term plasticity.

## Results

### Characterization of GIN neurons

**Figure 1:**
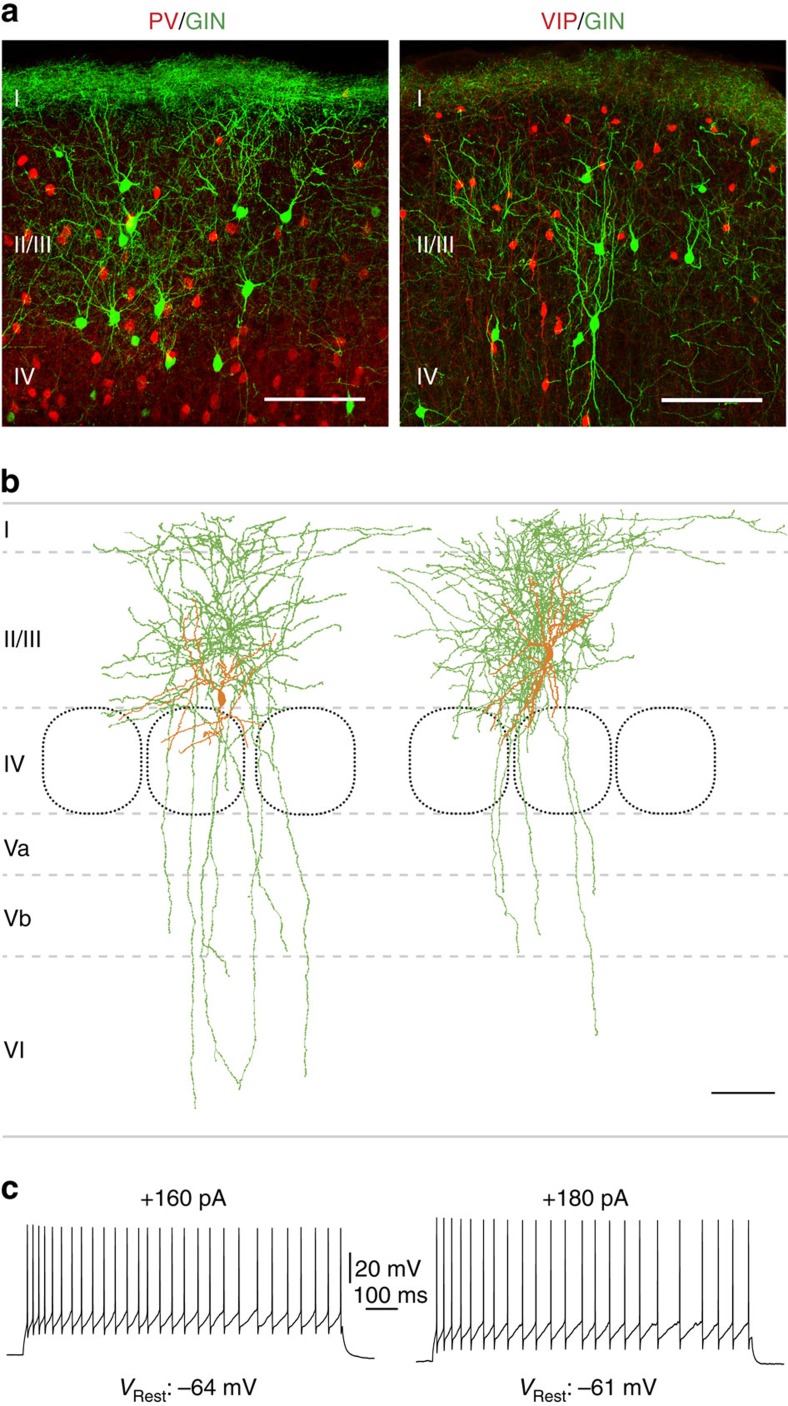
LII/LIII GIN cells show typical characteristics of Martinotti cells. (**a**) Fluorescent staining of triple transgenic mice (left: PV-cre::tdTomato::GIN, right: VIP-cre::tdTomato::GIN) used for the present experiments. In all, 50-μm-thick frontal sections from the barrel cortex are shown. PV or VIP cells, respectively, are labelled red and GIN cells green. Layers are indicated as I–IV. Scale bar, 100 μm. (**b**) Neurolucida reconstructions of LII/LIII biocytin-filled GIN cells. Somatodendritic compartments are shown in orange and axonal arborizations in green. Note the dense axonal branching in LI, which is characteristic for MC. Layers are indicated as I–VI. Scale bar, 100 μm. (**c**) Whole-cell current-clamp recordings of GIN cells shown in **b**. Depolarizing current injections caused an adapting firing pattern in these cells, as it is typical for MC.

To investigate inhibitory inputs to MC, we used two triple transgenic mouse lines, namely PV-cre::tdTomato::GIN and VIP-cre::tdTomato::GIN (Fig. 1a). The cre-knock in lines have recently been reported to be highly specific and sensitive mouse models^18^,^19^. Within the GIN line, green fluorescence protein (GFP)-expressing cells in cortical LII/LIII and LV were described as being almost exclusively MC^20^,^21^,^22^. In agreement with previous literature, we showed that, also in triple transgenic mice, these cells often display a multipolar or bitufted somato-dendritic configuration and, as their most prominent feature, dense axonal clustering in LI (Fig. 1b). Furthermore, MC show an adapting firing pattern during strong current injections (Fig. 1c). Our experimental data set contained 100 biocytin-labelled GIN cells, of which 82 could be morphologically recovered. Of these recovered neurons, 79 possessed extensive axonal arborizations in LI. In three cases, the ascending axon was cut off before reaching LI. Eight well-preserved GIN cells were fully reconstructed (Supplementary Figs 1 and 2). The dendrites were primarily located in LII/LIII (Supplementary Fig. 1b). Axonal density, however, peaked in LI as well as in LII/LIII (Supplementary Fig. 1b). Therefore, we will use the term MC in the following text to refer to GFP-expressing cells.

### MC are inhibited by local PV- and VIP-expressing cells

As mentioned above, PV and VIP cells form the main IN subpopulations besides MC, which are not considered to interact with each other^1^,^6^. Accordingly, they are likely candidates for providing inhibitory inputs to MC. To test this hypothesis, we expressed channelrhodopsin 2 (ChR2) in PV and VIP cells throughout the entire cortical depth of S1 by viral transfection. In order to control for the specificity of the input population, we recorded from ChR2-transduced PV and VIP IN (PV: 11 cells, 5 mice; VIP: 8 cells, 4 mice). They show their subgroup-specific firing patterns and morphology (Supplementary Fig. 3a–c,e–g). Additionally, we controlled for adequate ChR2 expression levels. Indeed, optogenetic stimulation of these cells caused depolarizations sufficient to fire action potentials (APs) (Supplementary Fig. 3d,h–k).

**Figure 2:**
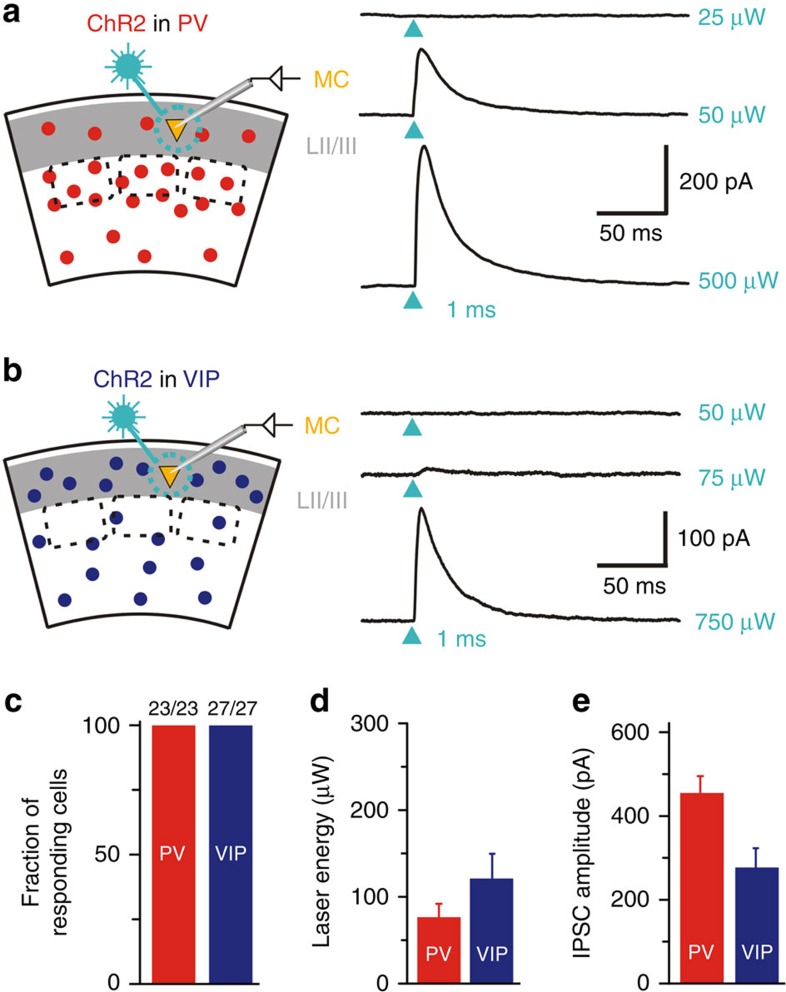
PV and VIP cells reliably target MC in LII/LIII of S1. (**a**,**b**) Left: schematic of recording configuration for photostimulation of ChR2-expressing PV or VIP cells while recording from LII/LIII MC. Right: Examples of photostimulation-induced inhibitory postsynaptic currents (IPSC). Arrowheads indicate photostimulation (473 nm laser, 1 ms) of PV interneurons (**a**) or VIP interneurons (**b**) at three different intensities (subthreshold, threshold and 10 × threshold). (**c**) Proportion of MC responding to photostimulation of PV and VIP cells. In both experimental designs, the success rate was 100%. This indicates that each MC receives inhibitory input from PV and VIP cells. (**d**) Threshold laser energy to elicit IPSC in MC by photostimulation of PV and VIP cells. Note the same range (mean±s.e.m) for both groups. (**e**) Mean±s.e.m. of IPSC amplitudes (at 10 × threshold level) in MC for each group (PV: *n*=16, age P38–P60, VIP: *n*=18, age P35–53). Optical stimulation of the PV cell population tends to result in larger multicomponent IPSC amplitudes.

Light-induced activation of ChR2 in either PV or VIP IN reliably evoked inhibitory postsynaptic currents (IPSCs) in every MC tested (PV: 23 cells, 12 mice, age P38–P60; VIP: 27 cells, 14 mice, age P35–P53) (Fig. 2a–c). This indicates that each MC receives input from PV and VIP cells. The minimal laser energy required to elicit IPSCs was comparable for PV and VIP inputs (PV: 76.7±15.0 μW; VIP: 124.0±27.1 μW; mean±s.e.m.) (Fig. 2d). PV cells evoked multicomponent IPSC with apparently larger amplitudes (PV: 456.55±43.22 pA; VIP: 275.70±46.59 pA; mean±s.e.m.) (Fig. 2a,b,e).

By means of optogenetic stimulation, we could define presynaptic IN populations; however, their precise laminar location remained unclear. Thus we localized sources of monosynaptic inhibitory input to LII/LIII MC by focal photolysis of caged glutamate (Supplementary Fig. 4). The highest proportion (∼45%) of inhibitory fields was found in LII/LIII of the home column (*n*=10, 10 mice).

### PV and VIP cell inhibition differs in synaptic properties

**Figure 3:**
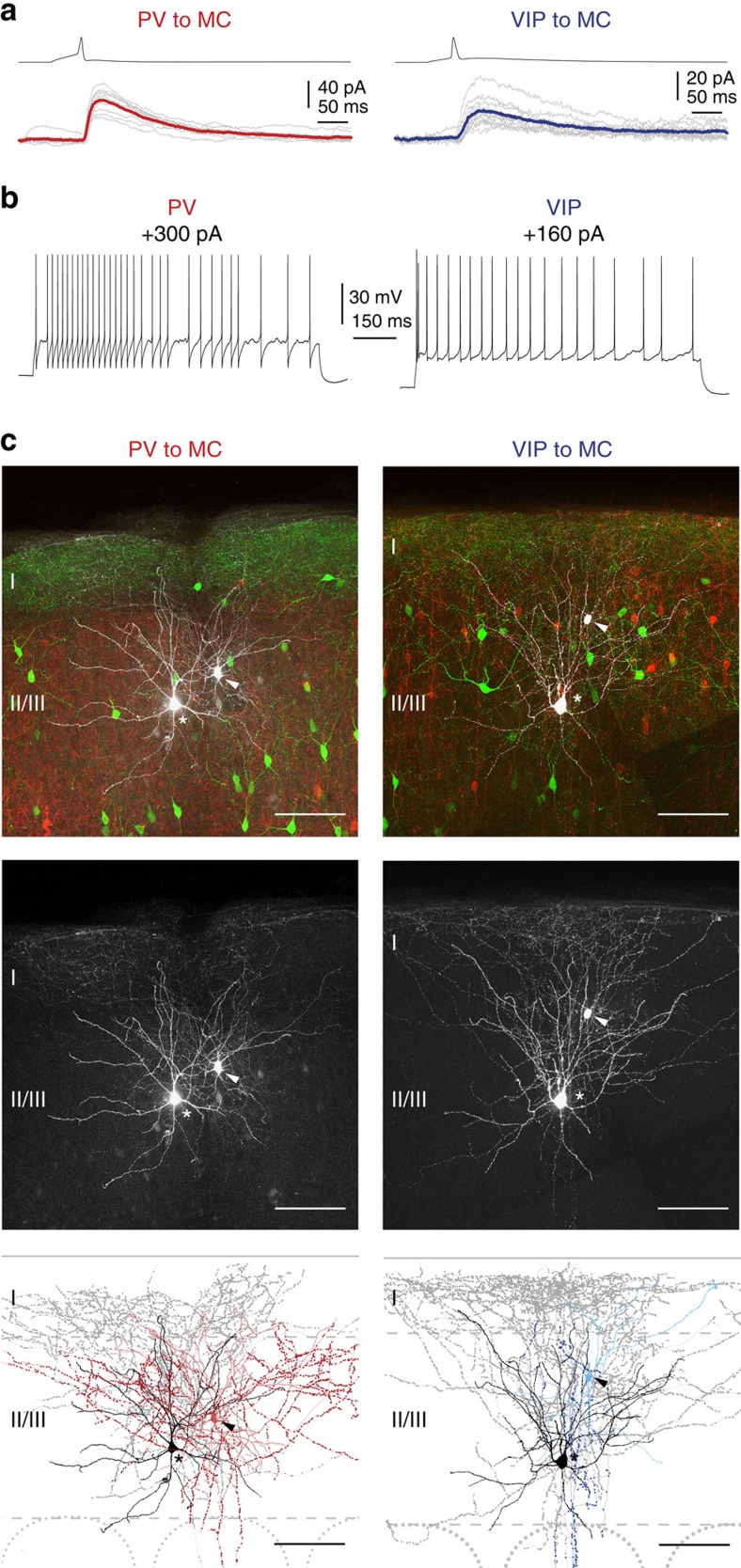
Electrophysiology and morphology of LII/LIII PV–MC and VIP–MC pairs. (**a**) Examples of connected pairs of presynaptic PV or VIP cells and postsynaptic MC in LII/LIII. The average of 10 individual IPSCs (grey traces, evoked by repetitive stimulation) is shown in colour (PV to MC: red, age P23; VIP to MC: blue, age P27). Presynaptic spikes reliably evoke IPSCs in both cases. (**b**) Whole-cell recordings of a presynaptic PV (left) and a VIP cell (right). During depolarizing current injections, the PV cell shows a fast spiking pattern, whereas the VIP cell shows an adapting firing pattern. (**c**) Staining of acute brain slices containing morphologically recovered and synaptically connected pairs as well as the corresponding Neurolucida reconstructions (left: PV to MC, right: VIP to MC). The connected cells are shown in white (pseudo-coloured). Asterisks mark MC somata, arrowheads somata of presynaptic cells. GIN cells are labelled green and the corresponding presynaptic population (PV or VIP) is labelled red (tdTomato-fluorescence). For clarity, connected cells are shown separately as grey-scale images in the middle. The reconstructed pairs are shown at the bottom. Soma and dendrites of GIN cells are labelled black and the corresponding axon grey. The recorded PV cell exhibits a multipolar dendritic morphology (light red) and a locally dense axon (red), as described for basket cells. The VIP cell shows an atypical tripolar dendritic configuration (light blue) and an axon (blue) descending towards the white matter. Complete reconstructions are displayed in Supplementary Fig. 4. Scale bars, 100 μm.

**Figure 4:**
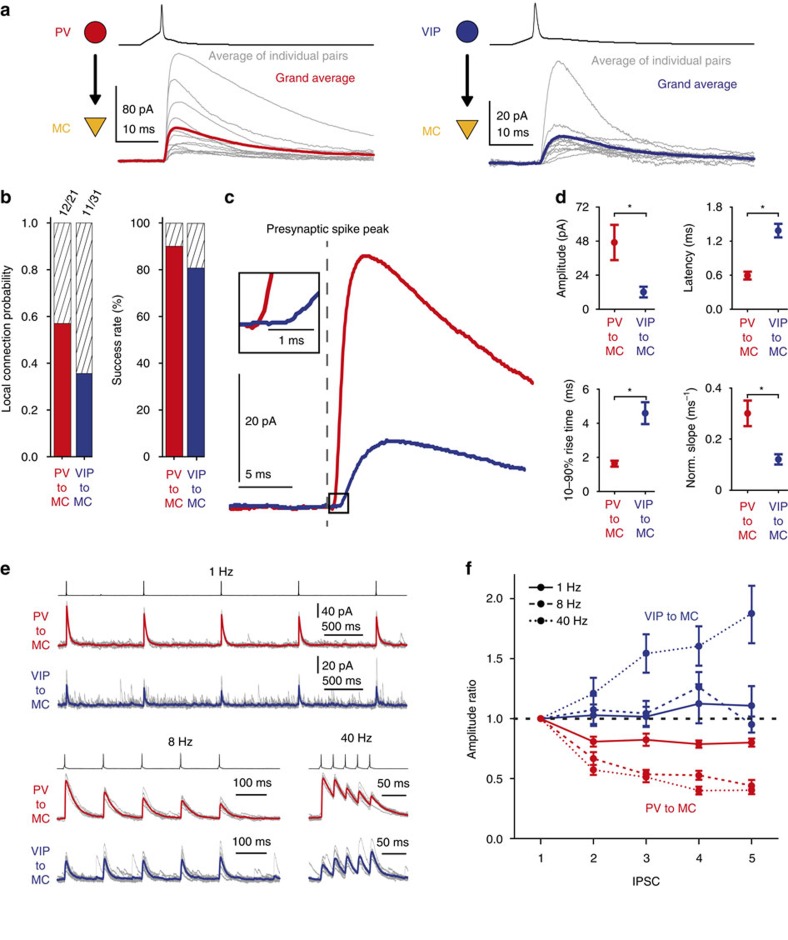
Unitary connections of PV and VIP cells onto MC differ in their elementary synaptic properties and short-term plasticity. (**a**) Grand average of unitary IPSCs (red: PV to MC, *n*=12, age P21–P36; blue: VIP to MC, *n*=11, age P21–P32) in MC in response to single spikes, repeatedly evoked in presynaptic IN. Averages of individual pairs are shown in grey. (**b**) Connection (left) and success rate of synaptic transmission (right) of the two different kinds of unitary connections. Note that the connection probability of PV cells (∼58%, 12/21) is substantially larger than the one of VIP cells (∼35%, 11/31). In connected pairs, synaptic transmission is highly reliable. (**c**) Overlay of grand averages (from **a**) aligned with respect to presynaptic spike peaks. IPSCs evoked by PV and VIP cells differ substantially in size and kinetics. For clarity, the boxed initial phase of both responses is shown at higher resolution as an inset. (**d**) Quantification of unitary IPSCs. Amplitude, latency, 10–90% rise time and normalized slope as fraction of amplitude per ms were analysed based on averages of each individual connected pair (PV to MC: red; VIP to MC: blue). Mean±s.e.m. was then calculated for each group separately. Asterisks indicate significant differences (*P*<0.05) for all those parameters. (**e**) Individual examples of averaged IPSCs in MC in response to trains of five spikes (1, 8 and 40 Hz) in a presynaptic IN (PV to MC: red trace; VIP to MC: blue trace). Individual traces are shown in grey. Quantification is shown in **f**. (**f**) Quantitative analysis of short-term plasticity at different frequencies (1 Hz: PV to MC, *n*=11; VIP to MC, *n*=11; 8 Hz: PV to MC, *n*=10; VIP to MC, *n*=11; 40 Hz: PV to MC, *n*=10; VIP to MC, *n*=10). Amplitude ratio (*n*th response/first response) of consecutive IPSCs plotted versus successive IPSCs. At the population level, PV to MC responses show synaptic depression under all stimulus conditions, whereas VIP to MC responses show no significant changes in amplitude at low frequencies but facilitate at 40 Hz. Values represent mean±s.e.m.

These findings indicated the presence of two discrete inhibitory inputs onto MC, namely, from PV and VIP cells, which are restricted to LII/LIII. Hence, we performed simultaneous recordings of putative presynaptic PV or VIP cells and postsynaptic MCs in this compartment. In accordance with previous results, we indeed found connected pairs of both types (age: PV MC: P21–P36; VIP MC: P21–P32; Fig. 3a). All PV and VIP cells matched their group-specific firing patterns and morphological characteristics, with PV cells being fast spiking multipolar cells and VIP cells being bipolar/bitufted cells (or partly modified variations thereof) of the adapting or irregular spiking type (Fig. 3b,c; Supplementary Fig. 2). We further analysed the elementary synaptic properties of connected pairs on a unitary level (Fig. 4; Supplementary Table 1a). The connection probability was higher for PV cells (∼58%, 12/21) than for VIP cells (∼35%, 11/31) (Fig. 4b). Based on the observation that each MC receives input from both of the two other types, as shown by optogenetic stimulation above, the differences in connection probability might be due to a higher degree of divergence of PV cell axonal projections onto MC, as proposed for PC targets as well^23^. Single presynaptic spikes reliably (PV to MC: ∼90%; VIP to MC: ∼80%) elicited unitary IPSCs in MC regardless of the type of presynaptic IN (Figs 3a and 4b). However, the average IPSC evoked by PV cells (*n*=12, 12 mice) showed significantly larger amplitude (PV to MC: 49.74±12.97 pA; VIP to MC: 12.13±3.57 pA; mean±s.e.m.), shorter latency (PV to MC: 0.60±0.07 ms; VIP to MC: 1.39±0.12 ms; mean±s.e.m.), shorter 10–90% rise time (PV to MC: 1.62±0.17 ms; VIP to MC: 4.59±0.64 ms; mean±s.e.m.) and higher normalized slope (PV to MC: 0.30±0.05 fraction of amplitude ms^−1^; VIP to MC: 0.12±0.02 fraction of amplitude ms^−1^; mean±s.e.m.) in comparison to VIP cell-evoked IPSCs (*n*=11, 9 mice) (Fig. 4c,d). Finally, in most of the cases we also probed for reciprocal connections. In six of the nine cases, PV cells were reciprocally connected with MC, whereas reciprocal connections between VIP cells and MC occurred only in one of the eight trials (Supplementary Fig. 5). We did not quantify these responses owing to differences in the intracellular solutions (see Methods section).

Information processing is subject to short-term dynamic changes in synaptic transmission^24^,^25^,^26^,^27^. Therefore, we triggered trains of presynaptic spikes at different frequencies (1, 8, 40 Hz) to investigate short-term plasticity for both types of pairs (Fig. 4e; for original data, see Supplementary Table 2a). The PV-to-MC connection showed significant depression in IPSC amplitude at all frequencies (1 Hz: *n*=11; 8 Hz: *n*=10; 40 Hz: *n*=10) (Fig. 4f; Supplementary Table 3a). Although the amount of depression differed from frequency to frequency, it was apparent and substantial (19.20±4.07%–42.75±4.11%; mean±s.e.m.) already in the second response in every case (Fig. 4f; Supplementary Table 3a). By contrast, repetitive firing in VIP cells neither caused synaptic depression nor obvious facilitation of inhibitory inputs to MC at lower frequencies (1 Hz: *n*=11; 8 Hz: *n*=11; 40 Hz: *n*=10). However, the amplitudes of IPSCs evoked by VIP cells consistently facilitated at 40 Hz beginning from the third response onwards (Fig. 4f; Supplementary Table 3a).

### PV-to-MC connections also exist in V1

The VIP-to-MC connection seems to be present in S1, as shown here and by Lee *et al*.^8^, and in the primary visual cortex (V1)^14^,^28^. However, it has been discussed recently whether or not the PV-to-MC connection does exist in V1 as well^6^,^29^,^30^. Accordingly, we performed paired recordings of PV cells and MC in LII/LIII of V1 (age: P27–P49; Supplementary Fig. 6a and Supplementary Table 1b). PV cells target MC with a connection probability of ∼35% (6/17) (Supplementary Fig. 6b). Similar to S1, PV cells reliably (∼97%) caused IPSCs in postsynaptic MC (Supplementary Fig. 6b). On average (mean±s.e.m.), the IPSCs had an amplitude of 50.68±13.70 pA, a latency of 0.68±0.08 ms, a 10–90% rise time of 1.77±0.20 ms and a normalized slope of 0.24±0.03 fraction of amplitude ms^−1^ (*n*=6 and 4 mice; Supplementary Fig. 6c). Concerning short-term plasticity, we observed synaptic depression already at 1-Hz stimulations (Supplementary Fig. 6d,e; for original data, see Supplementary Table 2b). This effect increased at higher frequencies of 8 and 40 Hz (Supplementary Fig. 6d,e; Supplementary Table 3b). Our data are thus in line with recent publications demonstrating that PV-to-MC connections are a frequent motif in V1^29^,^30^. As MC are only a subpopulation of SST-expressing IN^20^, this might explain previous discrepant results on PV-to-SST connections^6^.

## Discussion

The present study demonstrates that both VIP and PV cells target MC in LII/LIII of S1 and differ strongly in unitary synaptic properties as well as short-term plasticity. Although the VIP-to-MC connection has already been shown to exist in LII/LIII of different sensory cortical areas^7^,^8^,^14^,^29^,^30^, the PV-to-MC connection has largely been explored in LII/LIII of V1^29^,^30^. Here we show by combining optogenetics, glutamate uncaging, paired recordings and full morphological reconstruction that in S1 these two circuit motifs (VIP to MC and PV to MC) are fundamentally different.

An important finding is that these two inhibitory inputs onto MC differ substantially in IPSC amplitude, latency and kinetics. One mechanism possibly giving rise to such differences is divergent subcellular targeting. Owing to electrotonic spread, inputs located more distantly will be attenuated and slowed^31^. In addition, spread along the dendrite might account for the increase in latency. As VIP inputs in our sample are smaller in amplitude, slower in rise and relatively delayed, one might assume that VIP inputs onto MC are substantially more distal as compared with PV inputs. Indeed, such a separation of inhibitory inputs has been described for cortical PC^32^,^33^,^34^,^35^. Although dendrite-targeting inhibition is in a position to selectively control excitatory inputs, perisomatic inhibition exerts a global control of spike output. To our knowledge, however, there is no direct ultrastructural evidence for this targeting pattern on MC or any other kind of cortical IN as of yet. Alternatively, differences in the subunit composition of GABA_A_-receptors in MC could also account for the differences in unitary properties mentioned above^36^. Interestingly, these two alternatives are not mutually exclusive but might occur in parallel^37^.

Besides differences in size and kinetics, these two unitary connections also differ in short-term plasticity. Frequency-independent depression of PV cell input onto MC in LII/LIII in S1, as shown in the present report, is in line with previous observations that PV cell input shows short-term depression regardless of the type of postsynaptic cell in sensory cortical areas^38^. By contrast, reports on short-term plasticity of unitary VIP cell inputs in these areas are lacking. Although the frequency range tested here is in line with many related studies, it must be pointed out that both types of presynaptic neurons can fire at higher instantaneous frequencies. To our knowledge, the only study using presynaptic spike trains with very short interspike intervals reported substantial synaptic depression in fast spiking as well as non-fast spiking interneurons in adult rats over a wide range of interspike intervals except for 10–20 ms^39^. Short-term plasticity of PV inputs on MC might be described as phasic in comparison to the more tonic properties of VIP inputs. Nevertheless, even depressed PV inputs still exert a stronger influence at the soma than the corresponding VIP inputs. Considering that VIP cells might target distal dendrites, their true impact on dendritic input control would be much stronger and facilitation at high frequencies could be a dominant factor in controlling activity in the postsynaptic cell. By this means, VIP and PV cells may provide different spatial and temporal windows of opportunities^38^.

In the present study, we have shown that each MC in LII/LIII of the barrel cortex receives two different inhibitory inputs with divergent properties. Two separate input channels may, on the one hand, allow different sources of excitatory drive to control PC inhibition by MC but it may as well allow one single excitatory input to utilize different kinds of MC inhibition. Both types of MC-inhibiting IN share much of their excitatory input, namely, local LII/LIII PC and excitatory cells in LIV of the same column^40^,^41^,^42^. Recently, however, long-range connections from primary motor cortex have been described to preferentially target VIP cells in S1 (ref. 8). Accordingly, VIP cell-mediated inhibition of MC may integrate somatosensory and motor information, whereas PV cells are purely driven by somatosensory inputs. Besides glutamatergic input, VIP and PV cells also receive modulatory afferents. State-dependent cholinergic modulation may thereby selectively enhance and/or suppress activity in IN inhibiting MC^43^,^44^.

In summary, disinhibition of LII/LIII PC via MC is mediated by two distinct IN populations. As these inputs differ in strength, kinetics, short-term plasticity and possibly subcellular targeting, PV cells may induce a transient release from MC inhibition, whereas VIP cells may result in tonic disinhibition. Future studies will clarify whether tonic and phasic inhibition can be recruited by the same or different sources of inputs.

## Methods

### Animals

All experiments were performed in accordance with the German Law on the Protection of Animals. All animals used for breeding were obtained from Jackson Laboratory (Bar Harbor, USA) and kept under standard housing conditions. PV-cre (Pvalb^tm1(cre)Arbr^/J) or VIP-cre mice (VIP^tm1(cre)Zjh^) were crossbred with homozygous Ai9 mice (B6.Cg-Gt(ROSA)26Sor^tm9(CAG-tdTomato)Hze^/J) to obtain PV-cre/VIP-cre::tdTomato mice. These mice were further crossed with homozygous GIN mice (FVB-Tg(GadGFP)45704Swn) to create the triple transgenic mouse lines PV-cre::tdTomato::GIN and VIP-cre::tdTomato::GIN. Using these lines, PV-expressing (PV cell), VIP-expressing (VIP cell) and GIN cells in LII/LIII could be identified by their specific fluorescent label (PV/VIP cells: tdTomato fluorescence, GIN cells: GFP fluorescence). For optogenetic experiments, PV-cre (Pvalb^tm1(cre)Arbr^/J) or VIP-cre mice (VIP^tm1(cre)Zjh^) were crossbred with homozygous GIN mice (FVB-Tg(GadGFP)45704Swn) to create PV-cre::GIN and VIP-cre::GIN mouse lines.

### Slice preparation and chemicals

To obtain acute brain slices, juvenile mice of either sex (postnatal days 21–49) were deeply anaesthetized with isoflurane and decapitated. The brain was removed, the hemispheres separated and kept in cold (4 °C), oxygenated (Carbogen: 95% O_2_/5% CO_2_) preparation solution used for cutting (in mmol: 75 sucrose, 87 NaCl, 2.5 KCL, 0.5 CaCl_2_, 7.0 MgCl_2_, 26 NaHCO_3_, 1.25 NaH_2_PO_4_ and 10 glucose; pH: 7.4). Thalamo-cortical slices of 300 μm thickness from the mouse barrel cortex were prepared according to Porter *et al*.^45^ Coronal slices of 300 μm thickness were prepared from the primary visual cortex. Slices were incubated in oxygenated artificial cerebrospinal fluid (ACSF) (in mmol: 125 NaCl, 2.5 KCL, 2 CaCl_2_, 1 MgCl_2_, 26 NaHCO_3_, 1.25 NaH_2_PO_4_ and 25 glucose; pH: 7.4) at 32 °C for 30 min and later kept at room temperature until further processing.

### Electrophysiology and data acquisition

Slices were transferred to a submerged recording chamber (ACSF flow rate of 2 ml min^−1^ at 32 °C) in an upright microscope (Axio Examiner, Zeiss, Germany). For photostimulation, a 405 nm (DL-405, Rapp OptoElectronic, Wedel, Germany) or a 473 nm laser light (DL-473, Rapp OptoElectronic) was coupled via a 200 μm liquid fibre to the epifluorescence path of the microscope and guided into the × 40 objective. Whole-cell recordings from MC in LII/LIII of the barrel cortex and from PV or VIP cells were performed in current clamp as well as voltage clamp. Borosilicate patch pipettes (5–8 MΩ) contained a cesium-based intracellular solution (in mmol: 135 CsMeSO_4_, 5 CsCl, 2 MgCl_2_, 0.5 EGTA, 10 HEPES, 4 Mg-ATP, 0.3 Na-GTP, 10 Na-phosphocreatine phosphate; pH: 7.4) for GIN cell recordings during optogenetics, uncaging experiments and paired recordings. We used a potassium-based solution (in mmol: 135 K-gluconate, 5 KCl, 0.5 EGTA, 10 HEPES, 4 Mg-ATP, 0.3 Na-GTP, 10 Na-phosphocreatine phosphate; pH: 7.4) for PV or VIP cells in all experiments and GIN cells during experiments performed in current clamp. In case of paired recordings, we recorded from nearby (distance: 25–200 μm) PV and VIP cells. Intracellular solutions contained 0.3–0.5% biocytin for subsequent morphological visualization. Depolarizing current pulses above rheobase were used to characterize firing patterns of PV, VIP and GIN cells during initial current clamp recordings. To investigate IPSC, in all subsequent experiments, GIN cells were kept close to AMPA-receptor equilibrium potential in voltage clamp (*E*_AMPA_: ∼0 mV). This was carried out to increase the driving force for chloride, hence the amplitude of inhibitory postsynaptic currents, and to minimize contamination by excitatory postsynaptic currents. Data were acquired using a SEC-05 L amplifier (npi electronics, Tamm, Germany) in discontinuous mode with a switching frequency of 50 kHz. The signals were filtered at 3 kHz and digitized at 10–25 kHz using a CED Power 1401 interface (CED Limited, Cambridge, England). Data were collected, stored and analysed with Signal 5 (CED Limited, Cambridge, England).

### Focal photolysis of caged glutamate

As soon as stable whole-cell voltage clamp recordings of LII/LIII GIN cells (age P24–P34, either sex) were achieved (*V*_hold_=0 mV), focal photolysis of caged glutamate with a 405 nm laser light was carried out to activate presynaptic IN. The laser beam was focused on a 50 × 50 μm^2^ area on the plane of the brain slice. Caged glutamate (CNB-caged-L-glutamate, Molecular Probes, Carlsbad, USA) was added to the ACSF perfusion with a final concentration of 330 μM. To reduce detection errors of IPSCs, laser stimulus (6 ms duration) was repeated three times per field at an interval of 3 s. In principle, glutamate release could activate all types of neurons, which could lead to disynaptically evoked inhibitory inputs. We set up a series of calibration experiments to determine an energy level at which the laser, with its beam centred on the soma, generated spikes. Therefore, we performed sequential whole-cell patch clamp recordings, with potassium-based intracellular solution, of cortical neurons (PV, VIP, SST, GIN and excitatory cells) and induced spiking by glutamate uncaging. A laser energy level of ∼120 μJ triggered spikes in ∼86% of all types of inhibitory cells but in only ∼25% of excitatory cells located throughout all cortical layers (Supplementary Fig. 3e).

In subsequent mapping experiments, IPSCs were only accepted as stimulus evoked if their amplitude exceeded the mean baseline+3 s.d. of the baseline, they were detected in at least two out of three stimulus repetitions and they appeared within a 10 ms time window after stimulus offset. The laser was moved over an area covering three adjacent barrel-related columns (the middle one containing the recorded GIN cell) and the entire cortical depth, either from pia to white matter or vice versa. Scanning was carried out systematically along rows with alternating directions (50 μm per step, 10 s intervals) controlled by the Morgentau M1 software (Morgentau Solutions GmbH, Munich, Germany). Thus up to 364 different fields were stimulated without any intermittent gaps. In every slice containing a recorded GIN cell, layer and column borders were estimated from DAPI (4,6-diamidino-2-phenylindole) stainings and aligned with the scanned cortical area. Once individual fields were assignable to specific columns and layers, maps were created representing the average IPSC amplitude in fields containing sources of inhibitory input (inhibitory fields). These maps were then converted into binary ones by assigning a grey-scale value of 255 to each inhibitory field and the value 0 to the remaining fields (Supplementary Fig. 3f). In addition, the number of inhibitory fields was counted per layer and column. Individual binary maps were then aligned in relation to the barrel-like structure in LIV of the home column and converted into an average map depicting the confidence level for the position of inhibitory fields by means of a customized Matlab script (The MathWorks GmbH, Ismaning, Germany).

### Viral injection and optogenetics

To test whether PV and/or VIP cells target LII/LIII MC in the barrel cortex, we used adeno-associated viral vectors (AAV) for cre-dependent ChR2 expression in Cre-expressing interneurons. The AAV (pAAV-EF1a-double floxed-hChR2(H134R)-mCherry-WPRE-HGHpA) were custom manufactured by the ‘Viral Vectors Platform' of the DFG research unit and cluster of excellence CNMPB Goettingen and packaged in AAV-6 capsids. PV-cre::GIN or VIP-cre::GIN mice (postnatal days 18–27, either sex) were anaesthetized with 1–2% isoflurane (analgesia: Lidocaine s.c., metamizole p.o.) and positioned in a stereotactic frame. A small craniotomy (1–2 mm in diameter) was made with a dental drill (Osada Success 40, Osada, Tokyo, Japan) to expose the cortical surface. AAV were injected into the barrel cortex (anterior–posterior range: Bregma minus 1–2 mm, medial–lateral range: 2.5–3.5 mm) via a glass micropipette (25 μm inner diameter) connected to a Toohey Spritzer Pressure System IIe (Toohey Company, Fairfield, NJ, USA). The micropipette was positioned at 2–4 different locations, guided by surface blood vessel patterns and at 3 different depths (800, 500, 250 μm below the pial surface). Small amounts of virus in sterile phosphate-buffered saline (PBS; up to 150 nl) were injected by pressure application (3 psi, 250 ms pulse duration). The micropipette was withdrawn 10–15 min after pressure application. Finally, the animals were sutured and injected with carprofen (2.5 mg per 10 g body weight, Rymadyl, Pfizer, New York City, NY, USA). For postoperative care, animals were provided with wet food and metamizol (1.33 mg ml^−1^, Novaminsulfon-ratiopharm, Ratiopharm, Ulm, Germany) dissolved in drinking water. In accordance with animal care guidelines, metamizol was also applied a day before surgery.

Two-to-three weeks after viral transduction, acute brain slices were prepared and whole-cell patch clamp recordings of LII/LIII MC were performed as described above. Photostimulation (diameter: ∼100 μm) of ChR2-expressing cells was performed with a 473 nm laser light source (see above). The photostimulation (duration: 1 ms) was executed three times for three illumination intensities (subthreshold, threshold and 10 × threshold for IPSC occurrence in MC; range: 3–1,000 μW) at a minimum inter-stimulus interval of 5 s.

### Paired recordings

During paired recordings, presynaptic neurons (PV or VIP cells) remained at resting membrane potential (*V*_rest_) in current clamp. Postsynaptic GIN cells were kept at *V*_hold_=0 mV in voltage clamp. Consecutive brief current injections (5 ms per pulse, 20–650 pA, 10–20 sweeps, 10 s sweep interval) to presynaptic inhibitory neurons caused single spikes or a train of five spikes with frequencies of 1, 8 and 40 Hz (short-term plasticity experiments), which led to IPSCs in GIN cells.

All measurements were carried out on averages of individual sweeps. Prior to averaging, all individual IPSCs of a connected pair were aligned with respect to the spike peak of the presynaptic action potential. This was carried out to prevent disturbance of the average IPSC waveform owing to spike jitter. For responses from single spike stimulations, we analysed the following parameters: latency (time from presynaptic spike peak to IPSC onset), 10–90% rise time (time between 10% and 90% of IPSC peak amplitude), amplitude (difference from baseline to peak) and normalized slope of the ascending phase (average slope, determined by means of a least-square best fit, divided by the maximum amplitude) of the IPSC.

Short-term plasticity was tested by applying trains of presynaptic spikes. Here we only measured the peak amplitudes of the average IPSCs and calculated the response ratio for each IPSC relative to the amplitude of the first response (*n*th response/first response). Consecutive IPSCs overlapped only during 40 Hz stimulations. To measure the amplitude of single responses in this case, the decay phase of the preceding IPSC was exponentially fitted. This fit was extrapolated to baseline level. Response amplitude was then calculated as the difference between the peak of the response and the fit value at that point in time.

### Staining

To visualize biocytin-filled neurons as well as GFP-, mCherry- and tdTomato-expressing cells, slices were processed as follows. Slices were rinsed with PBS and incubated with primary antibodies (rabbit anti-RFP, 1:500, Rockland, Limerick, PA, USA; goat anti-GFP, 1:2,000, Abcam, Cambridge, UK) in blocking solution (0.25% bovine serum albumin, 10% normal donkey serum and 0.5% Triton X-100, pH 7.6, in PBS) for 48–72 h at 4 °C.Then they were rinsed in PBS (5 × ), followed by 4 h of secondary antibody incubation at room temperature, rinsed in PBS (6 × ) and stained by DAPI (1:1,000, Molecular Probes, Carlsbad, CA, USA). Secondary antibodies used were donkey anti-goat AF488 (1:500, Invitrogen, Carlsbad, CA, USA), donkey anti-rabbit AF546 (1:500) and streptavidin-conjugated AF633 (1:500). Slices were mounted in AquaPolyMount and fluorescent images were taken using a Leica SP2 confocal microscope ( × 40 immersion objective; voxel size: 0.18 × 0.18 × 0.80 μm^3^), controlled by the Vision4d software (Arivis AG, Unterschleißheim, Germany) in order to acquire and stitch multiple, predefined tiles.

### Reconstruction of biocytin-filled neurons

Fluorescently labelled neurons with consistently intense staining of neurites and no obvious truncation of main processes were reconstructed by acquiring large XYZ-stacks with a confocal microscope (Leica SP2; equipped with Arivis software) and subsequently loading these stacks into Neurolucida Workstation (MBF Bioscience, Colchester, VT, USA). Dendritic processes were distinguished from axonal structures by their diameter, fine structure and branching pattern.

To superimpose multiple reconstructions and show the distribution of neuronal processes as a population average, reconstructed neurons were registered into one file using defined layer borders (according to Prönneke *et al*.^18^) as a reference, somata aligned at the same vertical level and all fibers plotted as one binary image. This was filtered using a Gaussian filter with a comparable radial sigma (25 at 300 dpi) for all structures and a colour look-up-table ranging from cold (blue and green for white to light grey) to warm colours (yellow and red for dark grey to black) was applied to the resulting grey-scale image. This was then merged with the original black and white image and resulted in heat maps visualizing areas of the highest density of dendritic and axonal trees.

### Statistics

For statistical comparisons, Mann–Whitney rank-sum tests were used. Results were given as *P* values. *P*<0.05 was interpreted as significantly different. Mean±s.e.m. are given for all other values, if not stated otherwise.

### Digital illustrations

Confocal image stacks were exported as maximum intensity projections and stored as TIFF files. Image brightness and contrast images were adjusted using the Photoshop software (Adobe, Dublin, Ireland).

### Data availability

The data that support the findings of this study are available from the corresponding author on request. Neurolucida reconstructions will be made available through Neuromorpho.org.

## Additional information

**How to cite this article:** Walker, F. *et al*. Parvalbumin- and vasoactive intestinal polypeptide-expressing neocortical interneurons impose differential inhibition on Martinotti cells. *Nat. Commun.* **7**:13664 doi: 10.1038/ncomms13664 (2016).

**Publisher's note**: Springer Nature remains neutral with regard to jurisdictional claims in published maps and institutional affiliations.

## Supplementary Material

Supplementary Information Supplementary Figures 1 - 6 and Supplementary Tables 1 - 3

## References

[b1] Rudy B., Fishell G., Lee S. & Hjerling-Leffler J. Three groups of interneurons account for nearly 100% of neocortical GABAergic neurons. Dev. Neurobiol. 71, 45–61 (2011). 21154909 10.1002/dneu.20853 PMC3556905

[b2] Kepecs A. & Fishell G. Interneuron cell types are fit to function. Nature 505, 318–326 (2014). 24429630 10.1038/nature12983 PMC4349583

[b3] Staiger J. F., Möck M., Proenneke A. & Witte M. What types of neocortical GABAergic neurons do really exist? e-Neuroforum 6, 49–56 (2015).

[b4] DeFelipe J. . New insights into the classification and nomenclature of cortical GABAergic interneurons. Nat. Rev. Neurosci. 14, 202–216 (2013). 23385869 10.1038/nrn3444 PMC3619199

[b5] Isaacson J. S. & Scanziani M. How inhibition shapes cortical activity. Neuron 72, 231–243 (2011). 22017986 10.1016/j.neuron.2011.09.027 PMC3236361

[b6] Pfeffer C. K., Xue M., He M., Huang Z. J. & Scanziani M. Inhibition of inhibition in visual cortex: the logic of connections between molecularly distinct interneurons. Nat. Neurosci. 16, 1068–1076 (2013). 23817549 10.1038/nn.3446 PMC3729586

[b7] Pi H. J. . Cortical interneurons that specialize in disinhibitory control. Nature 503, 521–524 (2013). 24097352 10.1038/nature12676 PMC4017628

[b8] Lee S., Kruglikov I., Huang Z. J., Fishell G. & Rudy B. A disinhibitory circuit mediates motor integration in the somatosensory cortex. Nat. Neurosci. 16, 1662–1670 (2013). 24097044 10.1038/nn.3544 PMC4100076

[b9] Hangya B., Pi H. J., Kvitsiani D., Ranade S. P. & Kepecs A. From circuit motifs to computations: mapping the behavioral repertoire of cortical interneurons. Curr. Opin. Neurobiol. 26, 117–124 (2014). 24508565 10.1016/j.conb.2014.01.007 PMC4090079

[b10] Wang Y. . Anatomical, physiological and molecular properties of Martinotti cells in the somatosensory cortex of the juvenile rat. J. Physiol. 561, 65–90 (2004). 15331670 10.1113/jphysiol.2004.073353 PMC1665344

[b11] Silberberg G. & Markram H. Disynaptic inhibition between neocortical pyramidal cells mediated by Martinotti cells. Neuron 53, 735–746 (2007). 17329212 10.1016/j.neuron.2007.02.012

[b12] Gentet L. J. . Unique functional properties of somatostatin-expressing GABAergic neurons in mouse barrel cortex. Nat. Neurosci. 15, 607–612 (2012). 22366760 10.1038/nn.3051

[b13] Adesnik H., Bruns W., Taniguchi H., Huang Z. J. & Scanziani M. A neural circuit for spatial summation in visual cortex. Nature 490, 226–231 (2012). 23060193 10.1038/nature11526 PMC3621107

[b14] Fu Y. . A cortical circuit for gain control by behavioral state. Cell 156, 1139–1152 (2014). 24630718 10.1016/j.cell.2014.01.050 PMC4041382

[b15] Letzkus J. J. . A disinhibitory microcircuit for associative fear learning in the auditory cortex. Nature 480, 331–335 (2011). 22158104 10.1038/nature10674

[b16] Ascoli G. A. . Petilla terminology: nomenclature of features of GABAergic interneurons of the cerebral cortex. Nat. Rev. Neurosci. 9, 557–568 (2008). 18568015 10.1038/nrn2402 PMC2868386

[b17] Markram H. . Interneurons of the neocortical inhibitory system. Nat. Rev. Neurosci. 5, 793–807 (2004). 15378039 10.1038/nrn1519

[b18] Prönneke A. . Characterizing VIP neurons in the barrel cortex of VIPcre/tdTomato mice reveals layer-specific differences. Cereb. Cortex 25, 4854–4868 (2015). 26420784 10.1093/cercor/bhv202 PMC4635925

[b19] Taniguchi H. . A resource of Cre driver lines for genetic targeting of GABAergic neurons in cerebral cortex. Neuron 71, 995–1013 (2011). 21943598 10.1016/j.neuron.2011.07.026 PMC3779648

[b20] Ma Y., Hu H., Berrebi A. S., Mathers P. H. & Agmon A. Distinct subtypes of somatostatin-containing neocortical interneurons revealed in transgenic mice. J. Neurosci. 26, 5069–5082 (2006). 16687498 10.1523/JNEUROSCI.0661-06.2006 PMC2020857

[b21] Fanselow E. E., Richardson K. A. & Connors B. W. Selective, state-dependent activation of somatostatin-expressing inhibitory interneurons in mouse neocortex. J. Neurophysiol. 100, 2640–2652 (2008). 18799598 10.1152/jn.90691.2008 PMC2585405

[b22] McGarry L. M. . Quantitative classification of somatostatin-positive neocortical interneurons identifies three interneuron subtypes. Front. Neural Circuits 4, 12 (2010). 20617186 10.3389/fncir.2010.00012 PMC2896209

[b23] Packer A. M. & Yuste R. Dense, unspecific connectivity of neocortical parvalbumin-positive interneurons: a canonical microcircuit for inhibition? J. Neurosci. 31, 13260–13271 (2011). 21917809 10.1523/JNEUROSCI.3131-11.2011 PMC3178964

[b24] Markram H., Gupta A., Uziel A., Wang Y. & Tsodyks M. Information processing with frequency-dependent synaptic connections. Neurobiol. Learn. Mem. 70, 101–112 (1998). 9753590 10.1006/nlme.1998.3841

[b25] Fioravante D. & Regehr W. G. Short-term forms of presynaptic plasticity. Curr. Opin. Neurobiol. 21, 269–274 (2011). 21353526 10.1016/j.conb.2011.02.003 PMC3599780

[b26] Citri A. & Malenka R. C. Synaptic plasticity: multiple forms, functions, and mechanisms. Neuropsychopharmacology 33, 18–41 (2008). 17728696 10.1038/sj.npp.1301559

[b27] Markram H. & Tsodyks M. Redistribution of synaptic efficacy between neocortical pyramidal neurons. Nature 382, 807–810 (1996). 8752273 10.1038/382807a0

[b28] Karnani M. M. . Opening holes in the blanket of inhibition: localized lateral disinhibition by VIP interneurons. J. Neurosci. 36, 3471–3480 (2016). 27013676 10.1523/JNEUROSCI.3646-15.2016 PMC4804006

[b29] Jiang X. . Principles of connectivity among morphologically defined cell types in adult neocortex. Science 350, aac9462 (2015). 26612957 10.1126/science.aac9462 PMC4809866

[b30] Karnani M. M. . Cooperative subnetworks of molecularly similar interneurons in mouse neocortex. Neuron 90, 86–100 (2016). 27021171 10.1016/j.neuron.2016.02.037 PMC4961215

[b31] Spruston N., Jaffe D. B., Williams S. H. & Johnston D. Voltage- and space-clamp errors associated with the measurement of electrotonically remote synaptic events. J. Neurophysiol. 70, 781–802 (1993). 8410172 10.1152/jn.1993.70.2.781

[b32] Thomson A. M. & Bannister A. P. Interlaminar connections in the neocortex. Cereb. Cortex 13, 5–14 (2003). 12466210 10.1093/cercor/13.1.5

[b33] Kubota Y. . Functional effects of distinct innervation styles of pyramidal cells by fast spiking cortical interneurons. elife 4, 07919 (2015). 10.7554/eLife.07919 PMC4518632 26142457

[b34] Tamas G., Somogyi P. & Buhl E. H. Differentially interconnected networks of GABAergic interneurons in the visual cortex of the cat. J. Neurosci. 18, 4255–4270 (1998). 9592103 10.1523/JNEUROSCI.18-11-04255.1998 PMC6792813

[b35] Hajos F., Zilles K., Schleicher A. & Kalman M. Types and spatial distribution of vasoactive intestinal polypeptide (VIP)-containing synapses in the rat visual cortex. Anat. Embryol. (Berl) 178, 207–217 (1988). 3414975 10.1007/BF00318224

[b36] Bacci A., Rudolph U., Huguenard J. R. & Prince D. A. Major differences in inhibitory synaptic transmission onto two neocortical interneuron subclasses. J. Neurosci. 23, 9664–9674 (2003). 14573546 10.1523/JNEUROSCI.23-29-09664.2003 PMC6740477

[b37] Thomson A. M. & Jovanovic J. N. Mechanisms underlying synapse-specific clustering of GABA(A) receptors. Eur. J. Neurosci. 31, 2193–2203 (2010). 20550567 10.1111/j.1460-9568.2010.07252.x

[b38] Ma Y., Hu H. & Agmon A. Short-term plasticity of unitary inhibitory-to-inhibitory synapses depends on the presynaptic interneuron subtype. J. Neurosci. 32, 983–988 (2012). 22262896 10.1523/JNEUROSCI.5007-11.2012 PMC3291714

[b39] Thomson A. M., West D. C., Hahn J. & Deuchars J. Single axon IPSPs elicited in pyramidal cells by three classes of interneurones in slices of rat neocortex. J. Physiol. 496, 81–102 (1996). 8910198 10.1113/jphysiol.1996.sp021667 PMC1160826

[b40] Holmgren C., Harkany T., Svennenfors B. & Zilberter Y. Pyramidal cell communication within local networks in layer 2/3 of rat neocortex. J. Physiol. 551, 139–153 (2003). 12813147 10.1113/jphysiol.2003.044784 PMC2343144

[b41] Helmstaedter M., Staiger J. F., Sakmann B. & Feldmeyer D. Efficient recruitment of layer 2/3 interneurons by layer 4 input in single columns of rat somatosensory cortex. J. Neurosci. 28, 8273–8284 (2008). 18701690 10.1523/JNEUROSCI.5701-07.2008 PMC6670569

[b42] Porter J. T. . Properties of bipolar VIPergic interneurons and their excitation by pyramidal neurons in the rat neocortex. Eur. J. Neurosci. 10, 3617–3628 (1998). 9875341 10.1046/j.1460-9568.1998.00367.x

[b43] Jones B. E. Activity, modulation and role of basal forebrain cholinergic neurons innervating the cerebral cortex. Prog. Brain Res. 145, 157–169 (2004). 14650914 10.1016/S0079-6123(03)45011-5

[b44] Lee M. G., Hassani O. K., Alonso A. & Jones B. E. Cholinergic basal forebrain neurons burst with theta during waking and paradoxical sleep. J. Neurosci. 25, 4365–4369 (2005). 15858062 10.1523/JNEUROSCI.0178-05.2005 PMC6725118

[b45] Porter J. T., Johnson C. K. & Agmon A. Diverse types of interneurons generate thalamus-evoked feedforward inhibition in the mouse barrel cortex. J. Neurosci. 21, 2699–2710 (2001). 11306623 10.1523/JNEUROSCI.21-08-02699.2001 PMC6762510

