# Supplementary material for: Parvalbumin- and vasoactive intestinal polypeptide-expressing neocortical interneurons impose differential inhibition on Martinotti cells
Source: Nat Commun. 2016 Nov 29;7:13664. doi: 10.1038/ncomms13664 (PMC5141346; doi:10.1038/ncomms13664)
Supplement: Supplementary Information — Supplementary Figures 1 - 6 and Supplementary Tables 1 - 3 [file ncomms13664-s1.pdf]

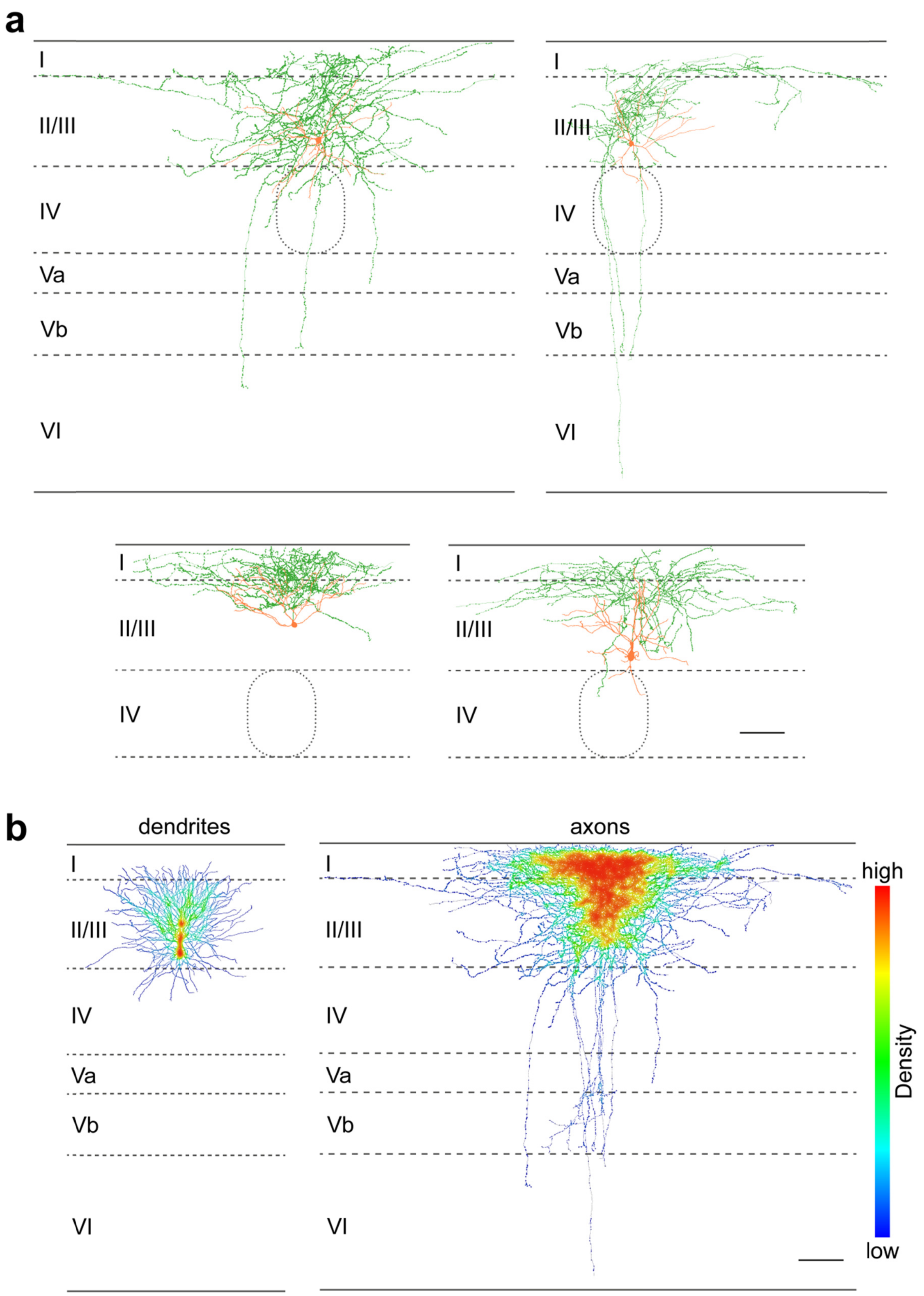
a
I
II/III
IV
Va
Vb
VI
b
dendrites
axons
high
Density
low

**Supplementary figure 1:** LII/III GIN-cells show morphological characteristics of MC

**(a)** Reconstructions of LII/III GIN-cells with somato-dendritic compartments in orange and axonal arborizations in green. Note the dense axonal branching in L I, which is indicative for MC. These data were taken from optogenetic experiments. Layers are labeled I-VI. Scale bar, 100 µm

**(b)** Vertical alignment of 8 reconstructed GIN-cells taken from **a** and **Supplementary Fig. 2** with respect to their soma location. The somato-dendritic alignment (left) indicates a multipolar configuration. Highest densities for axonal branches (right) can be found in LI and LII/III. Note that this branching pattern is representative for MC. Scale bar, 100 µm

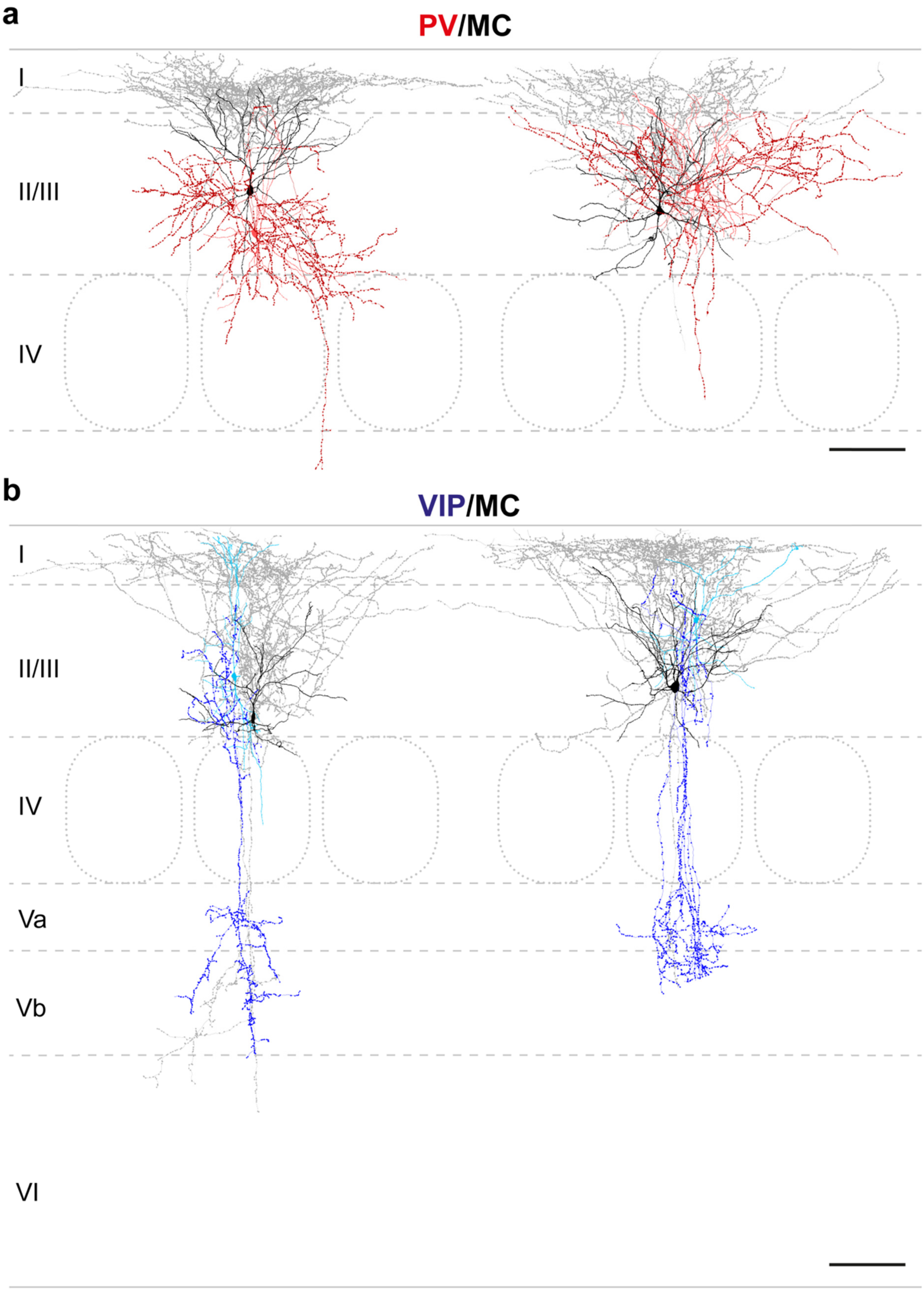
a
PV/MC
I
II/III
IV
b
VIP/MC
I
II/III
IV
Va
Vb
VI

**Supplementary figure 2:** Morphology of LII/III PV-MC and VIP-MC pairs

**(a)** Reconstructions of two PV-MC pairs. The soma and dendrites of the GIN-cells are labelled black and the axon in gray. Note the dense axonal arborization in LI. The PV-cells show a multipolar dendritic configuration (light red) and dense basket-cell like axonal distribution (red). Cortical layers are labeled I-IV. Scale: 100 μm

**(b)** Reconstructions of two VIP-MC pairs. GIN-cells are plotted with the same colors as in **a** (black: soma and dendrites, gray: axon). VIP-cells show a bipolar/bitufted dendritic configuration (light blue) and vertically descending axon (blue). Scale: 100 μm

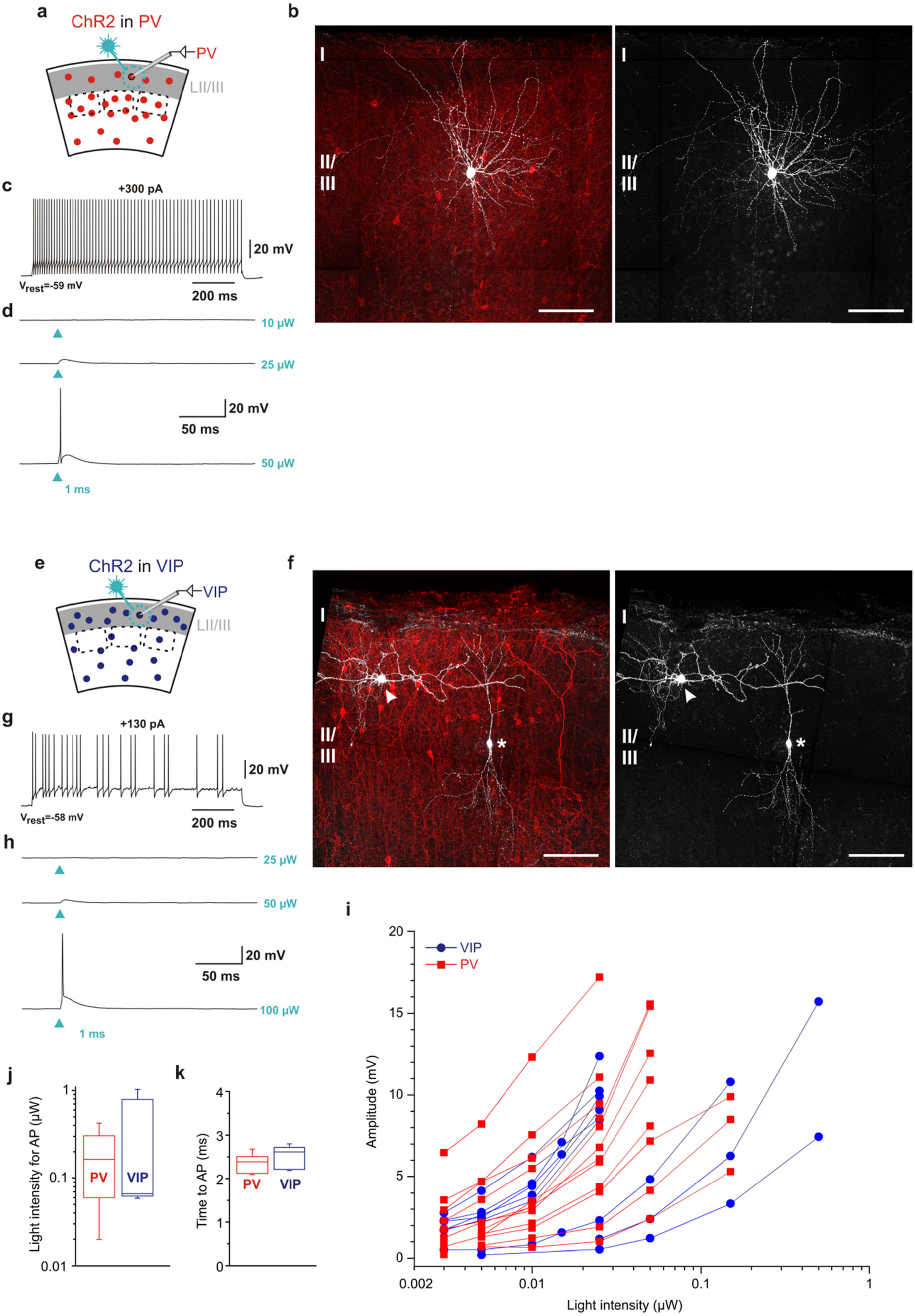
a
ChR2 in PV
PV
LII/III
b
I
II/
III
c
+300 pA
20 mV
Vrest=-59 mV
200 ms
d
10 µW
25 µW
20 mV
50 ms
50 µW
1 ms
e
ChR2 in VIP
VIP
LII/III
f
g
+130 pA
20 mV
Vrest=-58 mV
200 ms
h
25 µW
50 µW
20 mV
50 ms
100 µW
1 ms
i
VIP
PV
Amplitude (mV)
Light intensity (µW)
j
Light intensity for AP (µW)
PV
VIP
k
Time to AP (ms)
PV
VIP

**Supplementary figure 3:** Verification of the optogenetic approach: characterization of cell types and demonstration of ChR2-evoked activation

**(a, e)** Schematic of recording configuration and laser stimulation of transduced PV- **(a)** and VIP-cells **(e)** in LII/III.

**(b, f)** mCherry and biocytin-streptavidin labeling in acute brain slices. ChR2-transduced cells are labeled in red (mCherry-fluorescence). The biocytin-filled (and mCherry-positive) cells are shown in white (pseudo-colored). For clarity, recorded cells are shown separately as gray-scale images (right). The PV-cell labeled by streptavidin exhibits a multipolar dendritic morphology as described for basket cells **(b)**. The VIP-cell in LII/III (marked by asterisk) shows a bipolar dendritic configuration and an axon descending toward the white matter **(f)**. Note that the second labeled neuron (arrowhead) in **(f)** is an MC. This cell was recorded during subsequent experiments while activating a presynaptic population of VIP-cells. Scale bars, 100 µm

**(c, g)** Whole cell recording of mCherry-expressing cells, marked by asterisks in **b** and **f**. During depolarizing current injections, the PV-cell (age P42) shows a fast spiking firing pattern **(c)**, whereas the VIP-cell (age P44) shows an irregular firing pattern **(g)**.

**(d, h)** Examples of direct photostimulation. Arrowheads indicate photostimulation (473nm laser, 1ms) of the ChR2-transduced PV- (same as in **b** and **c**) and VIP-cell (same as in **f** and **(g)** at three different intensities (subthreshold, threshold, and spike evoking). Laser-stimulation leads to graded depolarizations, which finally evoke spikes. Note that photo-induced spiking was triggered in every recorded cell

**(i-k)** Quantification of ChR2-activation in transduced PV- and VIP cells (PV: n = 11, age P38-60; VIP: n = 8, age P35-53). Increased laser intensities lead to larger depolarization in both interneuron types **(i)**. The threshold of light intensity to induce AP firing is largely overlapping for PV- and VIP-IN (PV: median: 0.160 µW, 25% quartile: 0.054 µW, 75% quartile: 0.410 µW; VIP: median: 0.064 µW, 25% quartile: 0.059 µW, 75% quartile: 0.881

μW) **(j)**. Also the time to AP (from stimulus onset to AP peak) for both IN shows comparable values (PV: median: 2.39 ms, 25% quartile: 2.12 ms, 75% quartile: 2.51 ms; VIP: median: 2.67 ms, 25% quartile: 2.27 ms, 75% quartile: 2.78 ms) **(k)**.

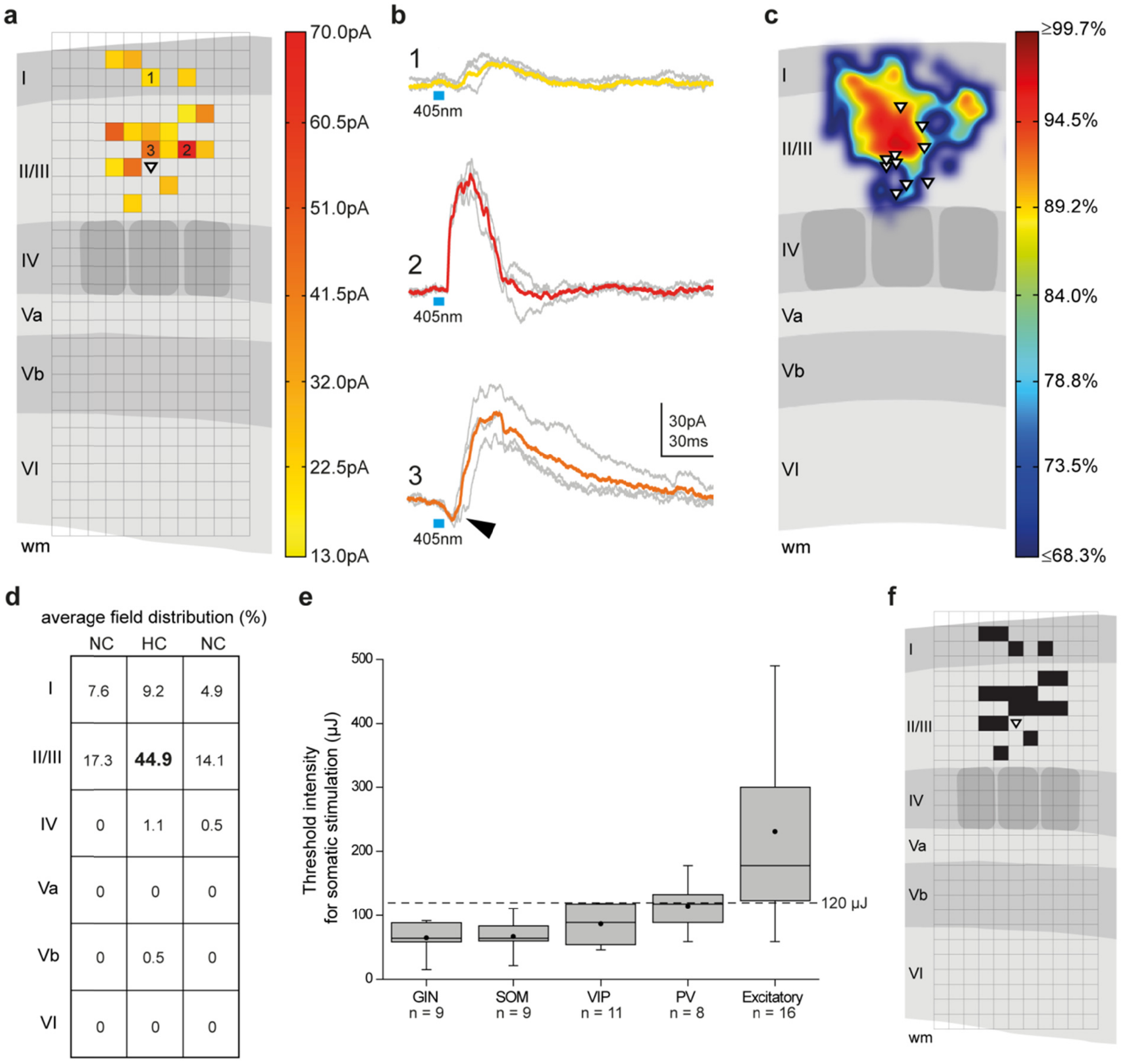


**Supplementary figure 4:** Glutamate uncaging reveals distribution of monosynaptic inhibitory input restricted to LII/III MC

**(a)** Example of a glutamate uncaging map of a MC (soma location:▽) in LII/III of S1. Monosynaptic inhibitory responses were evoked in color-coded fields. These fields were located only in LI and II/III. The color code depicts the average IPSC amplitude per field. IPSC amplitudes seem not to correlate with distance from MC soma. Average responses evoked from numbered fields (**1 - 3**) are shown in **b**. Layers are labeled I – VI, wm: white matter. Columns are indicated by schematic “barrels” in LIV **(a, c, f)**.

**(b)** Average (color-coded) and individual (gray) compound IPSCs in response to three successive laser-stimulations (blue bar: 6 ms, 405 nm) of fields marked in **a**. These examples show the typical range of amplitudes and waveforms. Note that example 3 consists of fast direct excitatory input (arrowhead) followed by strong monosynaptic inhibitory input. As the main focus was on the location of inhibitory inputs and not primarily on their precise amplitude, no measures to compute the true IPSC amplitude were taken in these cases.

**(c)** Average map (n = 10, age P24-34) illustrating the confidence level for the distribution of monosynaptic inhibitory input. Note that confidence levels ≥90 % are predominantly found in LII/III of the home column and neighboring columns, but also extend to some degree into LI. Confidence levels (≤68.3 % to ≥99.7 %) are color-coded.

**(d)** Table showing the layer- and column-specific distribution of inhibitory fields for the entire sample (n = 10). We calculated the relative proportion of inhibitory fields for all layers in the home column (HC) of the recorded MCs as well as the two adjacent neighboring columns (NC). Note that the highest percentage of inhibitory fields (~45%) is found in L II/III of the home column.

**(e)** Laser-calibration for glutamate uncaging. Box plots show the mean (black dot), the median, and the interquartile range of laser energy necessary to pass firing threshold for inhibitory (GIN-, SOM-, VIP-, and PV-cells) and excitatory cortical neurons at somatic locations. Whisker boundaries are the $10^{th}$ and $90^{th}$ percentile. The dashed line marks the laser energy used during uncaging experiments (120 µJ). Note that under these conditions, ~86% of INs, but only ~25% of excitatory neurons were driven to threshold.

**(f)** Example of a binary glutamate uncaging map. In binary maps, fields containing presynaptic INs are colored black regardless of the amplitude of the corresponding IPSC. The binary map here corresponds to the amplitude-coded example shown in figure 1a. These maps (n = 10) were used to calculate the distribution of inhibitory fields with respect to

layers and columns, and the average confidence level map shown in figure 1c. Layers are labeled I-VI, wm: white matter.

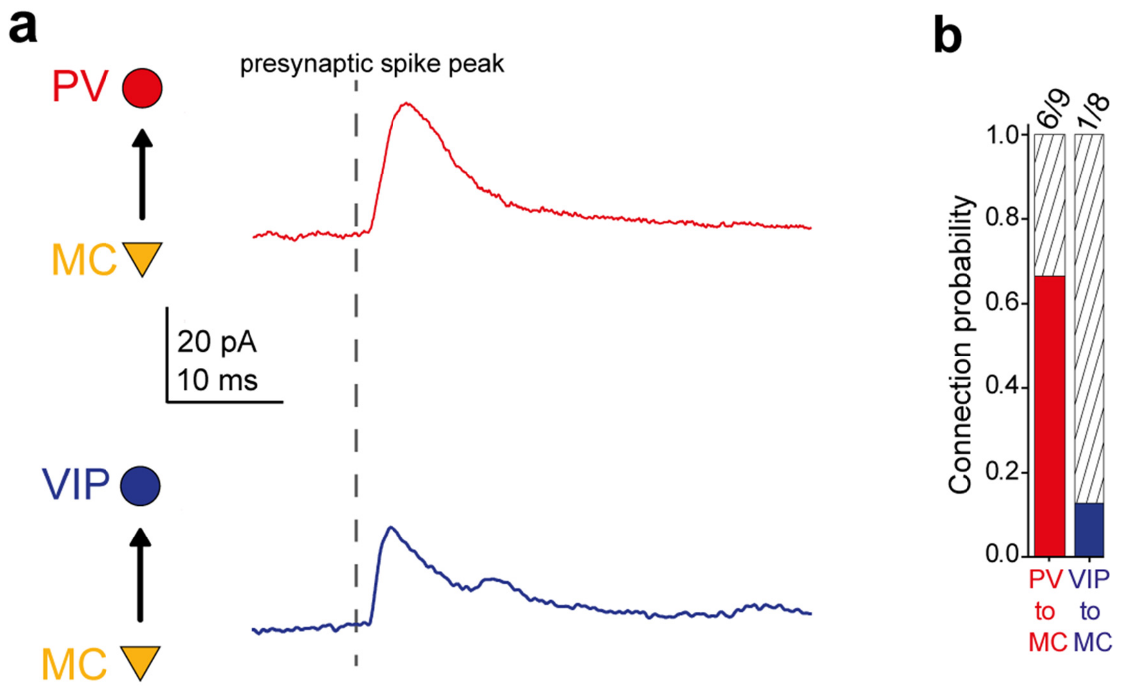


**Supplementary figure 5:** Reciprocal connections of PV-MC and VIP-MC pairs

**(a)** Average of unitary IPSC in one PV-cell (red; 10 traces, age P23) and in one VIP-cell (blue, 9 traces, age P25) evoked by repeatedly stimulating spikes in MC. IPSCs were aligned with respect to the presynaptic spike peak (dashed line) prior to averaging.

**(b)** PV-MC pairs are more likely to be reciprocally connected (~67%) than VIP-MC pairs (~12.5%).

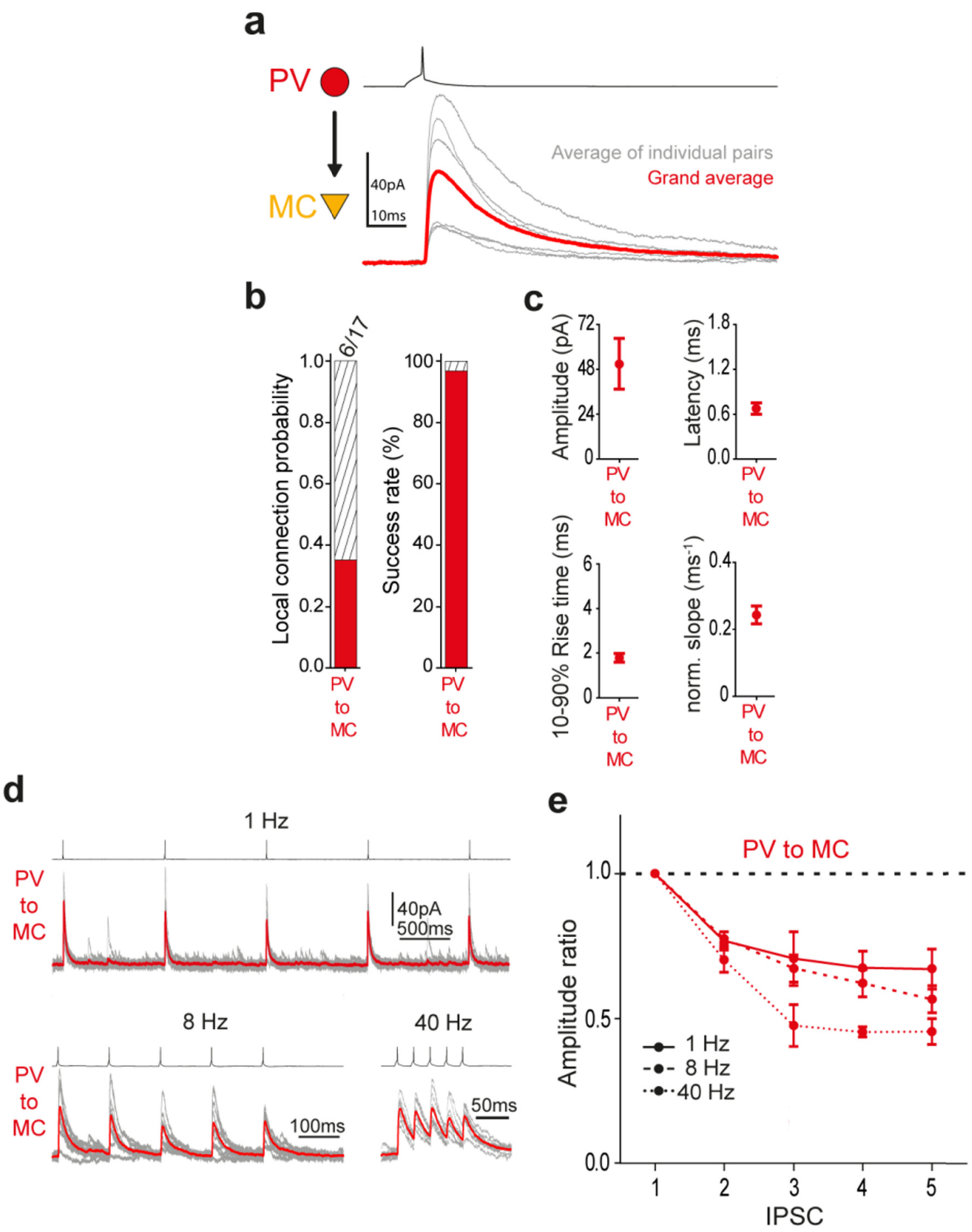
a
PV
MC
40pA
10ms
Average of individual pairs
Grand average
b
6/17
Local connection probability
1.0
0.8
0.6
0.4
0.2
0.0
PV to MC
Success rate (%)
100
80
60
40
20
0
PV to MC
c
Amplitude (pA)
72
48
24
0
PV to MC
Latency (ms)
1.8
1.2
0.6
0.0
PV to MC
10-90% Rise time (ms)
6
4
2
0
PV to MC
norm. slope (ms⁻¹)
0.4
0.2
0
PV to MC
d
1 Hz
PV to MC
40pA
500ms
8 Hz
40 Hz
PV to MC
100ms
50ms
e
PV to MC
1.0
0.5
0.0
Amplitude ratio
1 Hz
8 Hz
40 Hz
1
2
3
4
5
IPSC

**Supplementary figure 6:** Elementary synaptic properties and short-term plasticity of unitary connections of PV-cells and MC in primary visual cortex (V1)

**(a)** Grand average (red) of unitary IPSCs in MC in response to a single spike, repeatedly evoked in presynaptic PV-cells (n = 6, age P27-49). Averages of individual pairs are shown in gray.

**(b)** Connection probability (left) and success rate of synaptic transmission (right) of the recorded unitary connections. The connection probability of PV-cells with local MC was ~41%. In connected pairs, synaptic transmission was highly reliable.

**(c)** Quantification of unitary IPSCs. Amplitude, latency, 10-90% rise time, and normalized slope as fraction of amplitude per ms were analyzed based on averages of each individual connected pair. Afterwards, mean ± s.e.m was calculated.

**(d)** Individual examples of averaged IPSCs in MCs in response to trains of five spikes (1, 8 and 40 Hz) evoked in a presynaptic PV-cell. Individual traces are shown in gray. Quantification is shown in **e**.

**(e)** Quantitative analysis of short-term plasticity at different frequencies (1 Hz: n = 6; 8 Hz: n = 6; 40 Hz: n = 5). Amplitude-ratio ($n^{th}$ response/$1^{st}$ response) of consecutive IPSCs plotted versus successive IPSCs. At the population level, PV to MC responses show synaptic depression at all stimulus conditions. Values represent mean ± s.e.m.

**a**

**S1** — PV to MC

| Individual pair | Age | Amplitude | Latency | Rise time | Slope | Success rate |
|---|---|---|---|---|---|---|
| 1 | 31 | 114.90 | 0.43 | 0.89 | 0.45 | 100 |
| 2 | 36 | 11.33 | 1.02 | 2.21 | 0.18 | 90 |
| 3 | 32 | 21.32 | 0.47 | 1.69 | 0.26 | 100 |
| 4 | 33 | 26.44 | 0.64 | 2.96 | 0.13 | 90 |
| 5 | 34 | 151.43 | 0.52 | 1.34 | 0.27 | 100 |
| 6 | 42 | 82.98 | 0.44 | 1.41 | 0.20 | 100 |
| 7 | 23 | 60.90 | 0.36 | 1.32 | 0.37 | 100 |
| 8 | 26 | 40.87 | 0.60 | 1.27 | 0.33 | 100 |
| 9 | 31 | 22.88 | 0.48 | 0.82 | 0.73 | 79 |
| 10 | 32 | 16.59 | 0.51 | 1.90 | 0.16 | 75 |
| 11 | 21 | 11.53 | 1.07 | 2.11 | 0.11 | 60 |
| 12 | 28 | 35.68 | 0.63 | 1.50 | 0.40 | 100 |

VIP to MC

| Individual pair | Age | Amplitude | Latency | Rise time | Slope | Success rate |
|---|---|---|---|---|---|---|
| 1 | 21 | 5.41 | 1.75 | 9.08 | 0.05 | 100 |
| 2 | 21 | 7.57 | 2.10 | 7.57 | 0.08 | 100 |
| 3 | 24 | 6.36 | 1.45 | 5.27 | 0.06 | 80 |
| 4 | 26 | 9.91 | 1.10 | 4.10 | 0.07 | 89 |
| 5 | 32 | 7.75 | 1.54 | 3.28 | 0.12 | 70 |
| 6 | 27 | 5.24 | 0.66 | 5.71 | 0.06 | 60 |
| 7 | 25 | 46.37 | 1.16 | 2.37 | 0.25 | 100 |
| 8 | 27 | 7.16 | 1.40 | 3.09 | 0.10 | 83 |
| 9 | 27 | 17.19 | 1.26 | 2.58 | 0.29 | 74 |
| 10 | 24 | 10.49 | 1.68 | 3.35 | 0.13 | 67 |
| 11 | 24 | 10.01 | 1.15 | 4.14 | 0.14 | 67 |

**b**

**V1** — PV to MC

| Individual pair | Age | Amplitude | Latency | Rise time | Slope | Success rate |
|---|---|---|---|---|---|---|
| 1 | 41 | 92.72 | 0.60 | 1.62 | 0.20 | 100 |
| 2 | 40 | 68.03 | 0.69 | 1.44 | 0.22 | 100 |
| 3 | 49 | 22.45 | 0.36 | 1.64 | 0.30 | 100 |
| 4 | 49 | 80.63 | 0.68 | 1.59 | 0.32 | 100 |
| 5 | 27 | 19.39 | 0.86 | 1.60 | 0.28 | 80 |
| 6 | 27 | 20.89 | 0.87 | 2.75 | 0.13 | 100 |

**Supplementary table 1:** Quantification of unitary IPSC evoked in MC for each individual pair in S1 and V1.

**(a, b)** Table containing the age (postnatal day) of the mice and the corresponding averages of amplitude (pA), latency (ms), 10-90% rise time (ms), normalized slope (fraction of amplitude per ms) and success rate (%) from 10-20 trials per pair for **(a)** PV to MC connections (top row, 12 pairs) and VIP to MC connections (bottom row, 11 pairs) in S1 and **(b)** PV to MC connections (6 pairs) in V1.

**a**

**S1 — PV to MC**

| Individual pair | 1 Hz 1st | 1 Hz 2nd | 1 Hz 3rd | 1 Hz 4th | 1 Hz 5th | 8 Hz 1st | 8 Hz 2nd | 8 Hz 3rd | 8 Hz 4th | 8 Hz 5th | 40 Hz 1st | 40 Hz 2nd | 40 Hz 3rd | 40 Hz 4th | 40 Hz 5th |
|---|---|---|---|---|---|---|---|---|---|---|---|---|---|---|---|
| | Avg. amplitude of subsequent IPSCs (pA) | | | | | Avg. amplitude of subsequent IPSCs (pA) | | | | | Avg. amplitude of subsequent IPSCs (pA) | | | | |
| 1 | 104.46 | 78.83 | 80.37 | 92.49 | 81.40 | | | | | | | | | | |
| 2 | 14.74 | 11.96 | 13.87 | 10.76 | 14.65 | 16.03 | 10.57 | 11.85 | 9.51 | 10.36 | 14.15 | 9.92 | 9.55 | 7.85 | 7.99 |
| 3 | 17.58 | 15.80 | 15.07 | 15.01 | 17.60 | 18.74 | 13.53 | 8.77 | 11.41 | 6.00 | 21.43 | 12.42 | 14.90 | 5.50 | 9.07 |
| 4 | 128.77 | 106.93 | 100.68 | 96.01 | 95.17 | 128.27 | 83.72 | 65.41 | 57.29 | 47.95 | 131.82 | 64.83 | 40.89 | 44.77 | 33.22 |
| 5 | 98.03 | 65.41 | 69.42 | 66.57 | 74.50 | 92.65 | 69.25 | 51.60 | 42.79 | 40.06 | 93.48 | 56.79 | 42.82 | 40.02 | 34.64 |
| 6 | 9.70 | 5.46 | 6.80 | 6.69 | 8.28 | 7.75 | 8.41 | 5.58 | 5.50 | 4.92 | 8.28 | 5.82 | 5.79 | 3.87 | 3.70 |
| 7 | 56.70 | 44.43 | 40.06 | 45.92 | 45.01 | 56.44 | 32.82 | 29.33 | 25.62 | 19.52 | 52.22 | 29.62 | 24.78 | 20.16 | 22.42 |
| 8 | 53.48 | 41.29 | 30.32 | 47.37 | 37.92 | 52.40 | 30.39 | 27.16 | 28.89 | 24.40 | 48.10 | 33.48 | 24.06 | 24.04 | 17.97 |
| 9 | 9.59 | 7.82 | 11.55 | 5.95 | 7.61 | 16.29 | 9.79 | 6.10 | 5.97 | 9.89 | 10.18 | 6.64 | 5.05 | 4.85 | 5.34 |
| 10 | 10.55 | 11.51 | 9.79 | 9.68 | 6.89 | 13.86 | 5.96 | 7.59 | 9.33 | 2.86 | 12.29 | 4.20 | 5.50 | 4.03 | 4.90 |
| 11 | 37.63 | 33.87 | 33.83 | 31.78 | 27.35 | 43.34 | 26.10 | 17.13 | 17.67 | 16.56 | 45.46 | 17.48 | 16.59 | 12.13 | 11.18 |

**S1 — VIP to MC**

| Individual pair | 1 Hz 1st | 1 Hz 2nd | 1 Hz 3rd | 1 Hz 4th | 1 Hz 5th | 8 Hz 1st | 8 Hz 2nd | 8 Hz 3rd | 8 Hz 4th | 8 Hz 5th | 40 Hz 1st | 40 Hz 2nd | 40 Hz 3rd | 40 Hz 4th | 40 Hz 5th |
|---|---|---|---|---|---|---|---|---|---|---|---|---|---|---|---|
| | Avg. amplitude of subsequent IPSCs (pA) | | | | | Avg. amplitude of subsequent IPSCs (pA) | | | | | Avg. amplitude of subsequent IPSCs (pA) | | | | |
| 1 | 3.78 | 3.02 | 3.34 | 6.28 | 2.59 | 2.43 | 3.35 | 3.19 | 3.23 | 2.72 | | | | | |
| 2 | 6.32 | 5.31 | 6.50 | 3.59 | 3.48 | 5.63 | 4.35 | 2.69 | 4.27 | 2.87 | 5.64 | 4.72 | 6.87 | 6.79 | 10.16 |
| 3 | 3.63 | 2.85 | 2.98 | 3.64 | 3.83 | 2.60 | 2.84 | 4.16 | 4.28 | 2.49 | 3.73 | 5.10 | 3.46 | 4.45 | 4.99 |
| 4 | 7.58 | 9.87 | 8.29 | 7.17 | 7.86 | 5.23 | 7.02 | 7.76 | 6.89 | 6.25 | 4.43 | 6.04 | 5.98 | 6.76 | 8.38 |
| 5 | 9.46 | 7.97 | 8.76 | 8.61 | 10.34 | 12.32 | 12.44 | 11.27 | 10.45 | 10.02 | 11.16 | 10.20 | 14.96 | 12.22 | 11.39 |
| 6 | 3.86 | 6.04 | 6.44 | 9.60 | 9.73 | 6.49 | 4.57 | 5.34 | 8.24 | 5.35 | 4.19 | 9.06 | 10.44 | 9.14 | 14.35 |
| 7 | 43.62 | 44.03 | 37.49 | 45.98 | 50.43 | 39.20 | 43.20 | 40.25 | 54.45 | 50.83 | 37.08 | 52.46 | 68.34 | 66.30 | 64.94 |
| 8 | 2.68 | 4.11 | 2.81 | 2.84 | 4.31 | 4.05 | 2.92 | 2.85 | 4.66 | 2.77 | 3.83 | 2.93 | 7.06 | 5.43 | 6.03 |
| 9 | 13.19 | 13.73 | 11.25 | 12.41 | 11.24 | 5.28 | 10.45 | 6.61 | 11.34 | 5.58 | 11.68 | 12.53 | 16.82 | 14.94 | 14.00 |
| 10 | 4.70 | 4.31 | 6.19 | 5.28 | 4.58 | 4.67 | 3.03 | 3.74 | 3.83 | 4.55 | 6.41 | 7.84 | 11.14 | 16.89 | 18.20 |
| 11 | 8.03 | 6.08 | 6.35 | 4.39 | 6.09 | 4.40 | 4.51 | 5.15 | 4.34 | 4.81 | 5.51 | 5.01 | 5.24 | 7.75 | 7.02 |

**b**

**V1 — PV to MC**

| Individual pair | 1 Hz 1st | 1 Hz 2nd | 1 Hz 3rd | 1 Hz 4th | 1 Hz 5th | 8 Hz 1st | 8 Hz 2nd | 8 Hz 3rd | 8 Hz 4th | 8 Hz 5th | 40 Hz 1st | 40 Hz 2nd | 40 Hz 3rd | 40 Hz 4th | 40 Hz 5th |
|---|---|---|---|---|---|---|---|---|---|---|---|---|---|---|---|
| | Avg. amplitude of subsequent IPSCs (pA) | | | | | Avg. amplitude of subsequent IPSCs (pA) | | | | | Avg. amplitude of subsequent IPSCs (pA) | | | | |
| 1 | 78.07 | 64.59 | 54.75 | 66.57 | 58.48 | 59.63 | 46.63 | 34.30 | 40.10 | 41.34 | 54.84 | 35.97 | 38.59 | 28.30 | 31.14 |
| 2 | 93.69 | 72.72 | 63.99 | 73.03 | 66.27 | 87.58 | 67.99 | 69.42 | 47.20 | 45.61 | 70.02 | 65.94 | 51.83 | 51.66 | 49.60 |
| 3 | 27.02 | 17.45 | 23.22 | 18.14 | 21.41 | 20.51 | 14.19 | 14.98 | 10.75 | 11.11 | 26.48 | 14.89 | 9.89 | 11.47 | 9.31 |
| 4 | 86.17 | 66.86 | 64.02 | 57.15 | 69.67 | 81.53 | 71.46 | 64.68 | 67.67 | 54.56 | | | | | |
| 5 | 18.42 | 13.11 | 17.64 | 11.66 | 11.03 | 16.76 | 12.19 | 8.89 | 9.49 | 9.89 | 17.06 | 12.26 | 8.86 | 7.46 | 9.10 |
| 6 | 23.47 | 20.31 | 6.83 | 10.36 | 8.44 | 26.04 | 20.67 | 15.89 | 15.36 | 9.80 | 11.65 | 12.35 | 8.36 | 6.62 | 8.78 |

**Supplementary table 2:** Unitary IPSC amplitudes evoked in MC by presynaptic spike trains in PV- or VIP-cells.

**(a, b)** Summary of average amplitudes of the five subsequent IPSCs (1st to 5th) induced during train stimulations for all individual PV to MC connections (top row, 11 pairs) and VIP to MC connections (bottom row, 11 pairs) in S1 **(a)** and PV to MC connections (6 pairs) in V1 **(b)** and for the different stimulus frequencies (1, 8, and 40 Hz). Empty rows are due to incomplete recordings.

**a**

**S1 – PV to MC – 1 Hz**

| IPSC | 1 | 2 | 3 | 4 | 5 |
|---|---|---|---|---|---|
| 1 | | **<0.001** | **<0.001** | **<0.001** | **<0.001** |
| 2 | **<0.001** | | 0.809 | 0.690 | 0.886 |
| 3 | **<0.001** | 0.809 | | 0.546 | 0.702 |
| 4 | **<0.001** | 0.690 | 0.546 | | 0.778 |
| 5 | **<0.001** | 0.886 | 0.702 | 0.778 | |

**S1 – PV to MC – 8 Hz**

| IPSC | 1 | 2 | 3 | 4 | 5 |
|---|---|---|---|---|---|
| 1 | | **<0.001** | **<0.001** | **<0.001** | **<0.001** |
| 2 | **<0.001** | | **0.031** | **0.048** | **0.006** |
| 3 | **<0.001** | **0.031** | | 0.886 | 0.135 |
| 4 | **<0.001** | **0.048** | 0.886 | | 0.165 |
| 5 | **<0.001** | **0.006** | 0.135 | 0.165 | |

**S1 – PV to MC – 40 Hz**

| IPSC | 1 | 2 | 3 | 4 | 5 |
|---|---|---|---|---|---|
| 1 | | **<0.001** | **<0.001** | **<0.001** | **<0.001** |
| 2 | **<0.001** | | 0.323 | **0.004** | **0.004** |
| 3 | **<0.001** | 0.323 | | 0.052 | 0.058 |
| 4 | **<0.001** | **0.004** | 0.052 | | 0.954 |
| 5 | **<0.001** | **0.004** | 0.058 | 0.954 | |

**S1 – VIP to MC – 1 Hz**

| IPSC | 1 | 2 | 3 | 4 | 5 |
|---|---|---|---|---|---|
| 1 | | 0.262 | 0.726 | 0.726 | 0.726 |
| 2 | 0.262 | | 0.793 | 0.470 | 0.743 |
| 3 | 0.726 | 0.793 | | 0.555 | 0.743 |
| 4 | 0.726 | 0.470 | 0.555 | | 0.844 |
| 5 | 0.726 | 0.743 | 0.743 | 0.844 | |

**S1 – VIP to MC – 8 Hz**

| IPSC | 1 | 2 | 3 | 4 | 5 |
|---|---|---|---|---|---|
| 1 | | 0.262 | 0.726 | 0.262 | 0.726 |
| 2 | 0.262 | | 0.848 | 0.264 | 0.373 |
| 3 | 0.726 | 0.848 | | 0.200 | 0.702 |
| 4 | 0.262 | 0.264 | 0.200 | | 0.057 |
| 5 | 0.726 | 0.373 | 0.702 | 0.057 | |

**S1 – VIP to MC – 40 Hz**

| IPSC | 1 | 2 | 3 | 4 | 5 |
|---|---|---|---|---|---|
| 1 | | 0.443 | **0.017** | **<0.001** | **<0.001** |
| 2 | 0.433 | | 0.124 | 0.064 | **0.029** |
| 3 | **0.017** | 0.124 | | 0.910 | 0.272 |
| 4 | **<0.001** | 0.064 | 0.910 | | 0.372 |
| 5 | **<0.001** | **0.029** | 0.272 | 0.372 | |

**b**

**V1 – PV to MC – 1 Hz**

| IPSC | 1 | 2 | 3 | 4 | 5 |
|---|---|---|---|---|---|
| 1 | | **0.026** | **0.026** | **0.026** | **<0.001** |
| 2 | **0.026** | | 0.520 | 0.383 | 0.159 |
| 3 | **0.026** | 0.520 | | 0.987 | 0.575 |
| 4 | **0.026** | 0.383 | 0.987 | | 0.594 |
| 5 | **<0.001** | 0.159 | 0.575 | 0.594 | |

**V1 – PV to MC – 8 Hz**

| IPSC | 1 | 2 | 3 | 4 | 5 |
|---|---|---|---|---|---|
| 1 | | **<0.001** | **<0.001** | **<0.001** | **<0.001** |
| 2 | **<0.001** | | **0.029** | **0.013** | **0.001** |
| 3 | **<0.001** | **0.029** | | 0.600 | 0.057 |
| 4 | **<0.001** | **0.013** | 0.600 | | 0.136 |
| 5 | **<0.001** | **0.001** | 0.057 | 0.136 | |

**V1 – PV to MC – 40 Hz**

| IPSC | 1 | 2 | 3 | 4 | 5 |
|---|---|---|---|---|---|
| 1 | | **<0.001** | **<0.001** | **<0.001** | **<0.001** |
| 2 | **<0.001** | | **0.027** | **<0.001** | **0.004** |
| 3 | **<0.001** | **0.027** | | 0.770 | 0.813 |
| 4 | **<0.001** | **<0.001** | 0.770 | | 0.841 |
| 5 | **<0.001** | **0.004** | 0.813 | 0.841 | |

**Supplementary table 3:** P-values of statistical analysis of unitary IPSC amplitudes evoked in MC by presynaptic spike trains in PV- or VIP-cells.

**(a, b)** Tables containing p-values of the statistical analysis of normalized IPSC amplitudes for both PV to MC (top row) and VIP to MC connections (bottom row) in S1 **(a)** and for the PV to MC connection in V1 **(b)** and for the different stimulus frequencies (S1: 1 Hz: PV to MC: n = 11, VIP to MC: n = 11; 8 Hz: PV to MC: n = 10, VIP to MC: n = 11; 40 Hz: PV to MC: n = 10, VIP to MC: n = 11; V1: 1 Hz: PV to MC: n = 6; 8 Hz: PV to MC: n = 5; 40 Hz PV to MC: n = 5). Amplitude ratios ($n^{th}$-response/$1^{st}$-response) were calculated and tested for statistical differences. Significant differences ($P < 0.05$) are indicated by gray shading. Under all stimulus conditions, short-term plasticity was observed for the PV to MC connection. In response to 1 Hz stimulation there is a significant difference between the $1^{st}$ and the four subsequent IPSCs, the latter ones remaining at a similarly depressed amplitude level. Higher frequencies (8 and 40 Hz) induce further decrease in amplitude. Synaptic plasticity was absent at 1 Hz and 8 Hz stimulations for the VIP to MC connection. At 40 Hz stimulation, a significant facilitation of the IPSC amplitude was observed for the last three responses with respect to the $1^{st}$ IPSC.
